# Biphenyl as a Privileged Structure in Medicinal Chemistry: Advances in Anti-Infective Drug Discovery

**DOI:** 10.3390/molecules31071109

**Published:** 2026-03-27

**Authors:** Marilia Oliva Gandi, Rodolfo Rodrigo Florido França, Frederico Silva Castelo-Branco, Nubia Boechat

**Affiliations:** 1 Programa de Pós-Graduação em Farmacologia e Química Medicinal, Instituto de Ciências Biomédicas—ICB, Universidade Federal do Rio de Janeiro—UFRJ, Rio de Janeiro 21941-902, Brazil; mariliaolivagandi@gmail.com; 2 Laboratório de Síntese de Fármacos—LASFAR, Departamento de Síntese de Fármacos e Bioativos—DSFB, Instituto de Tecnologia em Fármacos—Farmanguinhos, Fundação Oswaldo Cruz—Fiocruz, Rio de Janeiro 21041-250, Brazil; rodolfo.franca@fiocruz.br (R.R.F.F.); frederico.castelo@fiocruz.br (F.S.C.-B.)

**Keywords:** biphenyl, drug discovery, medicinal chemistry, anti-infective, antifungal, antiviral, antibacterial, antiparasitic

## Abstract

The discovery of novel anti-infective agents is a continuous challenge in medicinal chemistry, particularly due to the rise in resistant fungal and viral strains. Within this context, the biphenyl subunit has been identified as a highly versatile privileged structure capable of interacting with diverse protein targets via hydrophobic and π-interactions. The purpose of this study is to review the pharmacological potential of biphenyl-based compounds, focusing on their application as anti-infective agents. We comprehensively analyzed recent literature and rational design strategies concerning biphenyl derivatives, examining structure-activity relationships, molecular docking insights, and structural optimizations aimed at enhancing both pharmacodynamics and pharmacokinetics. The reviewed studies demonstrate that incorporating biphenyl moieties yields compounds with potent antifungal and antiviral activities. Specifically, optimized biphenyl derivatives exhibit strong inhibitory effects against resistant Candida strains and crucial viral targets, including mutant variants of the HIV-1 reverse transcriptase and protease enzymes. Furthermore, strategic modifications, such as scaffold hopping and the introduction of specific substituents, successfully mitigated cytotoxicity and improved metabolic stability against cytochrome P450 enzymes. Biphenyl serves as a robust and adaptable scaffold for drug design. Its rational structural optimization provides a viable pathway to overcome drug resistance and develop effective, metabolically stable anti-infective therapeutics.

## 1. Introduction

The discovery of new molecules with biological activity has become increasingly complex, reflecting the multifactorial challenges inherent in modern drug-discovery pipelines. Advances in medicinal chemistry, high-throughput screening, and computational methodologies have expanded the chemical space; however, identifying lead compounds with adequate potency, selectivity, and pharmacokinetic properties remains a demanding and resource-intensive process. The term “privileged structure” was first introduced by Evans and colleagues in 1988 to describe molecular fragments capable of binding to multiple receptors. This definition suggests that these structures are inherently related to affinity, while selectivity can be achieved through subsequent structural optimizations carried out later in the molecule [1,2].

Aromatic subunits play a pivotal role in molecular recognition. Among these structural fragments, biphenyl is particularly noteworthy. In a screening based on NMR data carried out by Hajduk and colleagues, the biphenyl subunit demonstrated binding to almost half of the proteins tested. This suggests that the degree of flexibility of the aromatic bond may be responsible for these surprising results, given that the naphthyl subunit, for example, did not show the same results [3].

**Figure 1:**
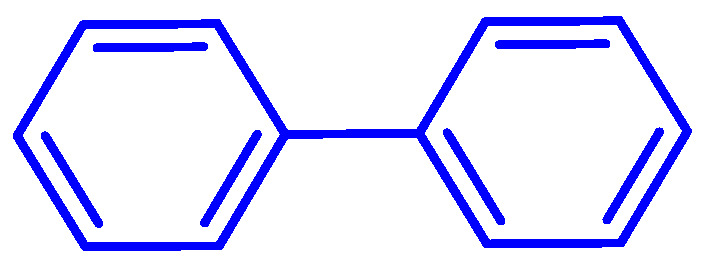
The chemical structure of biphenyl.

Biphenyl (Figure 1) is a structure that has been widely employed in the synthesis of a diverse array of compounds with a range of useful applications for humans. Initially, this subunit was utilized in the development of pesticides, known as PCBs (polychlorinated biphenyls). However, with the advent of advances in synthetic chemistry, a multitude of compounds with varying pharmacological activities have been created that contain this molecular fragment within their chemical structure [4].

**Figure 2:**
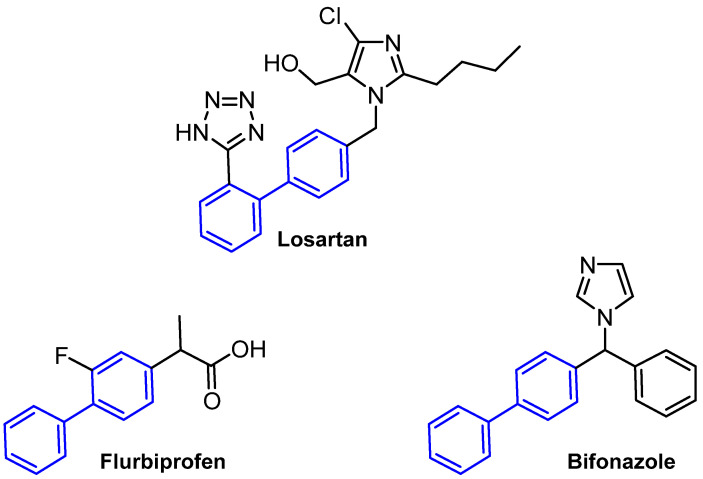
Examples of drugs that contain a biphenyl moiety. The biphenyl subunit is shown in blue.

Biphenyl is regarded as a privileged structure in the field of medicinal chemistry due to its capacity to form hydrophobic interactions with the binding pockets of target proteins. Moreover, this structure is capable of forming π-interactions with the amino acid residues tyrosine and tryptophan present in the targets. The biphenyl substructure demonstrated binding affinity for 45% of the assayed proteins during an NMR-based screening approach developed by Hajduk and co-workers [3]. Biphenyl is a prevalent structural element in numerous drugs, including the antihypertensive losartan, the anti-inflammatory flurbiprofen, and the antifungal bifonazole (Figure 2), as well as being a common constituent of natural products [4,5,6,7].

It is noteworthy that the biphenyl subunit is present in 4.3% of the drugs currently in clinical use and is employed in a range of therapeutic areas. The findings presented in the study by Hajduk et al. [3] also indicate that, despite the biphenyl subunit demonstrating affinity for a number of proteins, high levels of specificity can be achieved [3,8].

### Conformational Analysis and Thermodynamic Profiling of the Biphenyl Scaffold in Drug Design

The designation of the biphenyl subunit as a “privileged structure” in medicinal chemistry is linked to its unique conformational flexibility and dynamic thermodynamic profile [9]. Historically, drug design paradigms often oversimplified the biphenyl moiety, treating it as a static, planar, and purely hydrophobic shield. However, contemporary computational chemistry and high-resolution crystallographic analyses have demonstrated that the biphenyl system exists in a highly responsive dynamic equilibrium, governed by the interplay of resonance stabilization, steric hindrance, and electrostatic repulsions [9].

In the gas phase, the unsubstituted biphenyl core exhibits a preferred non-planar conformation, with the global energetic minimum corresponding to a dihedral angle (the torsion angle around the C1-C1′ inter-annular bond) of approximately 44.4° [9]. This geometry represents the thermodynamic compromise between the energetic penalty of disrupting the stabilizing π-π conjugation across the two aromatic rings (which favors a 0° coplanar state) and the intense steric repulsion between the proximal *ortho*-hydrogen atoms (which drives the rings toward a 90° orthogonal state) [9].

Extensive Conformational Energy Profile (CEP) analyses utilizing ab initio density functional theory (DFT) have established a definitive hierarchy of forces that drives the preferred conformation of biaryl systems within biological environments: steric repulsion > lone pair–lone pair repulsion > lone pair–fluorine repulsion > resonance stabilization [10,11]. Notably, resonance stabilization is the weakest determinant of the conformational energy profile; even the minimal steric footprint of unsubstituted *ortho*-hydrogens is sufficient to override resonance, leading to the characteristic twisted ground state [11]. However, when accommodated within the constrained microenvironment of a protein binding pocket, external packing forces and hydrophobic collapse can induce the molecule to adopt atypical coplanar or fully orthogonal geometries, provided the binding enthalpy overcomes the internal torsional strain penalty [10].

The most profound application of biphenyl conformational analysis in modern drug discovery is the active exploitation of “conformational locking” and atropisomerism [12]. The strategic introduction of bulky substituents—such as halogens (chlorine, bromine) or methyl groups—at the *ortho* positions (2, 2′, 6, 6′) drastically increases the rotational energy barrier around the chiral axis [12]. By employing this rationale, medicinal chemists can purposefully lock the biphenyl moiety into a specific dihedral conformation that acts as a precise topographical match for the target receptor’s three-dimensional architecture. This rigidification strategy dramatically reduces the entropic penalty typically associated with the binding of highly flexible ligands, exponentially increasing binding affinity and drastically enhancing target selectivity by preventing the ligand from binding to off-target biological macromolecules [12].

## 2. Biological Activities

### 2.1. Antifungal Activity

Fungal infections are a global threat to public health and are associated with a high mortality rate. The population is susceptible to a variety of superficial, subcutaneous, cutaneous and, in many cases, systemic infections caused by these opportunistic fungal pathogens, which are difficult to treat [13,14,15].

The fungal genera that pose the greatest threat to public health are *Cryptococcus*, *Candida*, *Aspergillus* and *Pneumocystis*. The increasing number of immunocompromised patients has led to an increase in the frequency and severity of these fungal infections [16,17].

Recurrent fungal infections are caused by strains that are resistant to available drugs, especially *Candida albicans* (*C. albicans*), the most common fungal pathogen. Therefore, the development of antifungal drugs is extremely important as the drugs available for the treatment of these infections are limited.

Zhao et al. developed a series of imidazole-biphenyl derivatives that showed excellent antifungal activity against *C. albicans* and *Cryptococcus neoformans* (*C. neoformans*), with minimum inhibitory concentrations (MICs) ranging from 0.25 µg/mL to 2 µg/mL. However, all compounds were inactive against *Aspergillus fumigatus* (*A. fumigatus*), encouraging structural optimization of these compounds [18].

**Figure 3:**
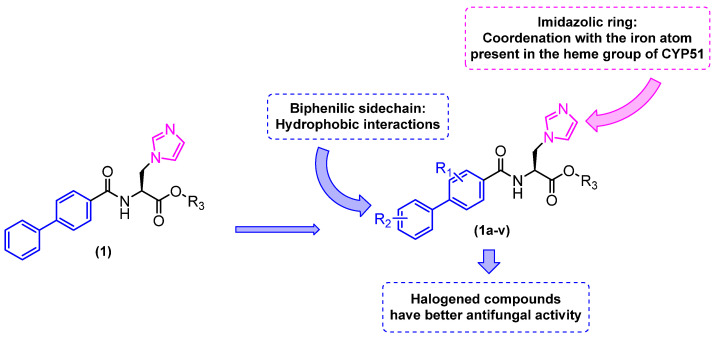
Design of new imidazole-biphenyl derivatives.

To improve activity against *A. fumigatus*, compound **1** was selected for a molecular docking study on the CYP51 enzyme of *A. fumigatus* (PDB: 4UYM), and based on the observed interactions, structural modifications were designed. This enzyme is involved in the biosynthesis of ergosterol in fungi and is one of the most clinically validated therapeutic targets. A total of 22 new imidazole biphenyl derivatives were designed and synthesized (Figure 3). The in vitro antifungal activities of these compounds were evaluated against the fungal pathogens *Candida albicans*, *Candida tropicalis*, *Aspergillus fumigatus*, and *Cryptococcus neoformans*. The MIC was determined using the broth microdilution test in 96-well plates. The antifungal drugs fluconazole (FCZ) and itraconazole (ITZ) were used as reference drugs (Table 1). The compounds demonstrated moderate to good antifungal activity, with compounds **1i**, **1j**, **1m–p**, and **1s–v** exhibiting the most pronounced inhibitory effects against strains of *Candida albicans* and *Candida tropicalis*, with minimum inhibitory concentration (MIC) values ranging from 0.03125 to 2 µg/mL. The compound **1m** exhibited remarkable efficacy among the compounds tested against the strains under investigation, demonstrating activity that was either superior or comparable to that of the reference drugs fluconazole and itraconazole (Table 1).

In view of the resistance issues associated with the first-line antifungals, the activity of compounds **1m**, **1o**, **1u** and **1v** was assessed against strains of *C. albicans* exhibiting resistance to fluconazole. Derivatives **1m** and **1v** demonstrated moderate activity against these strains, with respective MIC values of 2 and 8 µg/mL (Table 2).

Cytochrome P450 enzymes play a pivotal role in the metabolism of xenobiotics and have been linked to adverse reactions resulting from drug interactions [19]. An understanding of the metabolic stability of a novel drug candidate is a crucial aspect of the drug development process, as the pharmacokinetic properties of these compounds are significantly influenced by this parameter. It is estimated that approximately 75% of drugs are metabolized by cytochrome P450, primarily by the CYP3A4, CYP2D6, CYP2C9, CYP2C16, and CYP1A2 isoforms. Compounds with high metabolic rates may exhibit a short half-life, potentially leading to reduced efficacy or the necessity for a less convenient dosage regimen [20,21].

It is therefore of interest to evaluate the potential interactions of drug candidate compounds with these enzymes. The inhibitory potential of compounds **1m** and **1o** was evaluated against the primary cytochrome P450 isoforms (CYP1A2, CYP2C9, CYP2C19, CYP2D6, and CYP3A4). The results demonstrated that compound **1m** exhibited no inhibitory activity against the CYP1A2, CYP2C9, CYP2C19, and CYP2D6 isoforms. Additionally, it presented weak inhibition of CYP3A4 (IC_50_ = 4.05 µM), indicating a low affinity for these enzymes. Conversely, compound **1o** demonstrated a notable inhibitory effect against CYP3A4 with an IC_50_ value of 0.0429 µM. Regarding toxicity, compounds **1m** and **1o** were evaluated for their effects on mammalian cells (A549 and U87) using MTT assays and demonstrated IC_50_ values of less than 50 µM.

Medicinal chemistry plays a foundational role in the process of drug discovery and development. The planning of bioactive compounds should not be limited to considerations of pharmacodynamics; it is also essential to optimize pharmacokinetic parameters, which are crucial for ensuring the efficacy and safety of drugs.

**Figure 4:**
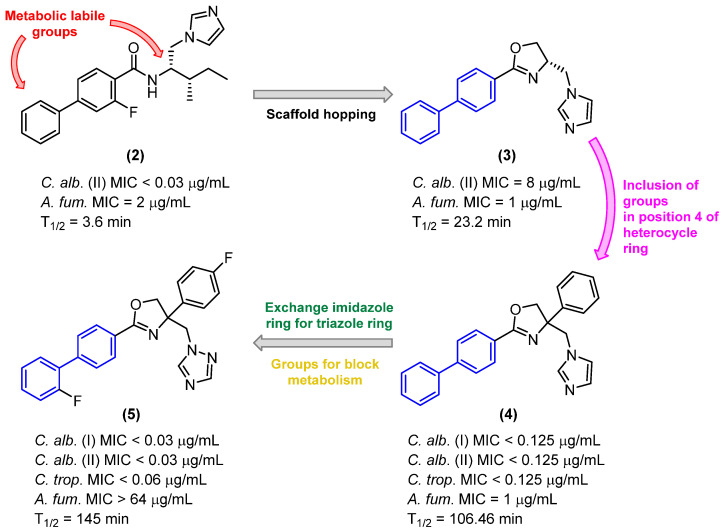
Design of new triazole biphenyl derivatives.

In a study published by Zhao et al. [22], biphenyl derivatives were developed which demonstrated high antifungal activity; however, these compounds exhibited low metabolic stability. Compound **2** was the most active member of the series, exhibiting an MIC value of less than 0.03 µg/mL. In the in vitro evaluation in liver microsomes, compound **2** demonstrated a half-life of 3.6 min, indicating low metabolic stability. This prompted the design of a new series of derivatives using the scaffold hopping strategy (Figure 4), with the aim of improving the pharmacokinetics of this compound.

The results indicate that the phenyl ring and the alkyl chain are the most favorable sites for metabolism (Figure 4). Accordingly, in order to mitigate biotransformation reactions in these regions, a series of strategies were implemented, including the reduction of lipophilicity, the elimination of the alkyl chain, and the incorporation of electroreductive substituents in the biphenyl subunit.

Replacing the amide with the dihydrooxazole heterocycle and removing the alkyl chain and fluorine atom from the biphenyl ring resulted in an increase in metabolic stability, as evidenced by the approximately six-fold increase in the half-life time of compound **3** (T_1/2_ = 23.2 min) relative to that of compound **2** (T_1/2_ = 3.6 min). However, a notable decline in antifungal efficacy was observed against both *C. albicans* and *A. fumigatus*. Subsequently, compound **3** was selected for further structural modifications with the objective of optimizing its antifungal activity and metabolic stability.

Zhao and colleagues [22] observed that the introduction of groups at position 4 of the dihydrooxazole ring (Figure 4) resulted in an increase in antifungal activity. Derivative **4** demonstrated an MIC value of less than 0.125 µg/mL against *C. albicans* (GIM2.194) and 32 µg/mL against *C. neoformans*. This indicates that it was at least 64 and 2 times more potent than derivative **3**. Furthermore, there was an increase in metabolic stability, with derivative **4** (T_1/2_ = 106.46 min) exhibiting a half-life approximately four times longer than that of **3** (T_1/2_ = 23.2 min).

To circumvent the potential oxidation reactions prompted by CYPs while preserving antifungal efficacy, electroreductive groups were incorporated into the biphenyl and phenyl rings, and the imidazole heterocycle was replaced with the triazole (Compound **5**, Figure 4).

A comparison of the results with the imidazole compound revealed that the triazole compound maintained antifungal activity against *C. albicans* and *C. tropicalis*, exhibiting excellent activity against the aforementioned strains with MIC values of 0.03 µg/mL and 0.06 µg/mL, respectively. Additionally, the triazole compound demonstrated moderate activity against *C. krusei* with an MIC value of 0.25 µg/mL. Furthermore, compound **5** demonstrated a notable enhancement in metabolic stability within human liver microsomes in vitro, exhibiting a half-life exceeding 145 min. The metabolic stability of this compound in human plasma in the presence of hydrolase enzymes was also assessed, and the findings indicated that derivative 5 exhibited a minimal rate of biotransformation by these enzymes after 120 min of incubation, with a half-life exceeding 4.8 h. This suggests that the compound possesses favorable plasma stability.

**Figure 5:**
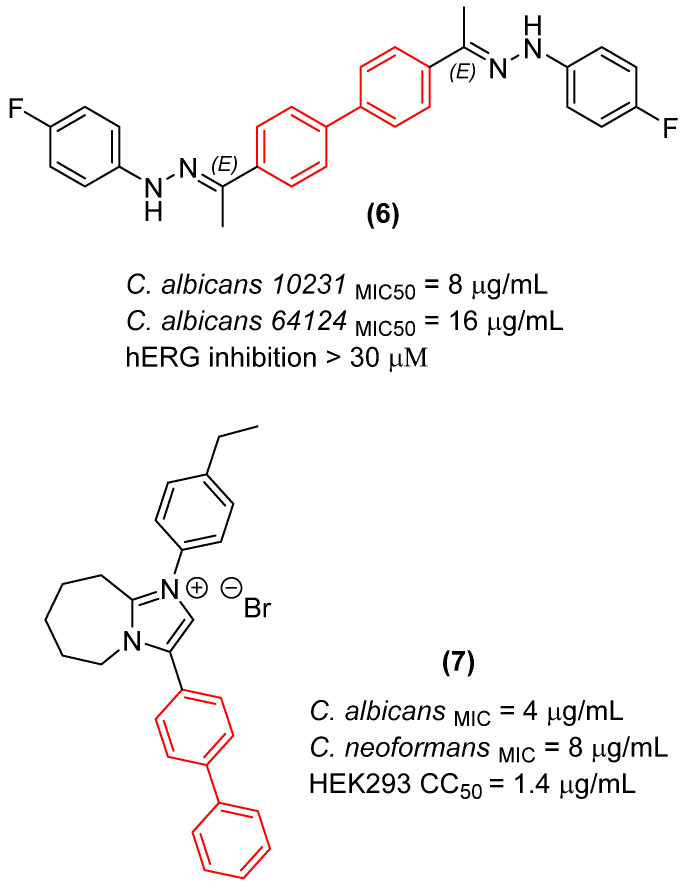
Pharmacological profiling of compounds **6** and **7**.

Recent structural optimization strategies have increasingly capitalized on the biphenyl core’s ability to act as a robust, conformationally restricted spacer. For instance, the development of N,N′-diaryl-bishydrazones built upon a strict biphenyl platform has yielded broad-spectrum antifungal agents with remarkable efficacy against both filamentous and non-filamentous fungi. The biphenyl linker in these scaffolds provides an optimal spatial distance between the terminal hydrazone pharmacophores. Although the exact molecular target of this class remains unconfirmed, this rigidification is hypothesized to enable simultaneous interactions with distinct hydrophobic sub-pockets within an unidentified fungal receptor. This structural rigidification is essential for minimizing the entropic penalty upon binding, which directly translates to the observed high potency and an exceptionally low hemolytic profile against mammalian cells. A critical structure-activity relationship (SAR) emerged regarding the substitution topology: while an unsymmetrical 3,4′-substitution pattern on the biphenyl core was generally favored for derivatives bearing a proton at the imine carbon, a symmetrical 4,4′-substitution was optimal for methyl-substituted derivatives. Pharmacological profiling of the lead candidate, compound **6** (Figure 5), demonstrated fungistatic action in time-kill kinetics and it proved to be more potent than voriconazole in resistant *C. albicans* strains (Figure 5). Crucially, the compound exhibited a favorable safety profile, inducing less murine erythrocyte hemolysis than voriconazole at comparable concentrations, while showing no evidence of mammalian cytotoxicity or hERG-mediated cardiotoxic liability [23].

Furthermore, Demchenko et al. [24] expanded the antifungal utility of the biphenyl moiety by synthesizing a library of novel 3-biphenyl-3H-imidazo[1,2-a]azepin-1-ium bromides. In these quaternary salts, the biphenyl unit acts as an extended, highly lipophilic tail that dramatically improves the antifungal spectrum. The optimized derivative (**7**, Figure 5) exhibited relevant polyvalent activity against *Cryptococcus neoformans* and *C. albicans*. The biphenyl group in this context facilitates deep insertion into the fungal cell membrane’s lipid bilayer, corroborating its role as a privileged structural anchor for membrane-active antifungal agents. Nonetheless, despite the absence of hemolytic activity, the compounds exhibited significant cytotoxicity against human embryonic kidney cells (HEK293), thereby compromising the selectivity of this series [24].

Additionally, a critical appraisal of such highly lipophilic, rigid platforms reveals inherent pharmacokinetic liabilities. The thermodynamic penalty of desolvating these rigid, highly hydrophobic structures during target binding remains a significant hurdle for in vivo translation. In the case of Demchenko’s quaternary salts, although the biphenyl tail maximizes membrane insertion, this non-specific hydrophobic anchoring raises critical questions regarding long-term selectivity and the potential for off-target accumulation in mammalian lipid-rich tissues over extended treatments. The inherent poor aqueous solubility driven by the unadulterated biphenyl core often necessitates complex formulation strategies. Future rational design must judiciously balance this strict lipophilicity with polar appendages to optimize oral bioavailability and metabolic stability, without compromising the structural integrity that confers their antifungal potency.

A deep analysis of the structure-activity relationships (SAR) among biphenyl-based antifungal agents reveals a pronounced dependence on the substitution pattern of the biaromatic core, which directly dictates the spatial trajectory of the attached pharmacophores. For instance, in bishydrazone derivatives, an unsymmetrical 3,4′-substitution pattern on the biphenyl platform consistently outperforms symmetrical 4,4′-arrangements. This 3,4′-geometry induces a specific kink in the molecular backbone. It is postulated that this geometry provides an optimal distance and vector for engaging multiple sub-pockets within the putative fungal target simultaneously, an interaction that is sterically hindered in the more linear 4,4′-isomers [23]. Furthermore, the electronic nature of the substituents on the terminal aryl rings synergizes with the biphenyl core. The incorporation of electron-withdrawing and lipophilic groups, such as para-fluoro or para-trifluoromethyl moieties, significantly improves the metabolic stability and target affinity. The fluorine atoms not only increase the overall lipophilicity—facilitating the penetration through the complex ergosterol-rich fungal membrane—but are also hypothesized to participate in multipolar interactions within the binding site of the uncharacterized target, while the rigid biphenyl core minimizes the entropic loss upon binding [23].

**Figure 6:**
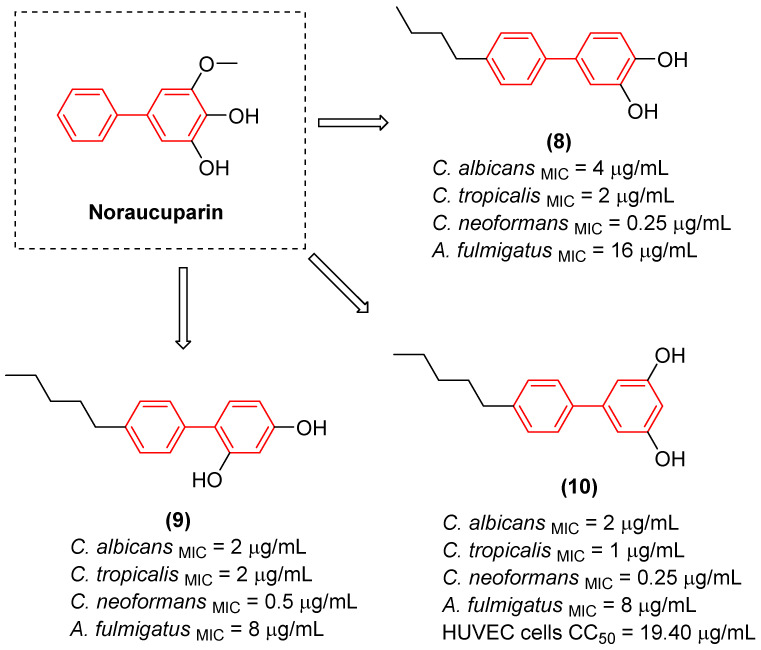
Pharmacological profiling of compounds **8**–**10**.

Beyond traditional synthetic screening, the structural inspiration derived from natural biphenyl phytoalexins has catalyzed the development of novel antifungal agents. Taking cues from noraucuparin (Figure 6), a naturally occurring biphenyl phytoantitoxin, Liu et al. [25] designed a series of new biphenyl derivatives aimed at combating invasive fungal infections. Through systematic modifications of the dual benzene rings, compounds such as **8**, **9**, and **10** (Figure 6) emerged as potent broad-spectrum agents. These optimized derivatives demonstrated minimum inhibitory concentrations as low as 0.25–16 µg/mL, representing an 8- to 256-fold potency enhancement over the parent noraucuparin. Notably, compound **10** exhibited efficacy comparable to amphotericin B against *Cryptococcus neoformans* and proved much less cytotoxic to Human Umbilical Vein Endothelial Cells (HUVEC cells), showing significant selectivity. Preliminary mechanistic studies highlighted that the biphenyl core acts as a critical lipophilic anchor, driving rapid fungicidal action through severe disruption of the fungal cell membrane and the inhibition of key infective factors, all while maintaining a favorable safety profile in mammalian cells [25].

Furthermore, the topographic flexibility of the biphenyl core has been critically evaluated in the design of antimicrobial peptidomimetic amphiphiles. Tague et al. systematically investigated how positional isomerism on a rigid biphenyl scaffold dictates the spatial arrangement of hydrophobic (isopentyl) and hydrophilic (cationic peptide) domains, profoundly shifting the antimicrobial spectrum [26].

**Figure 7:**
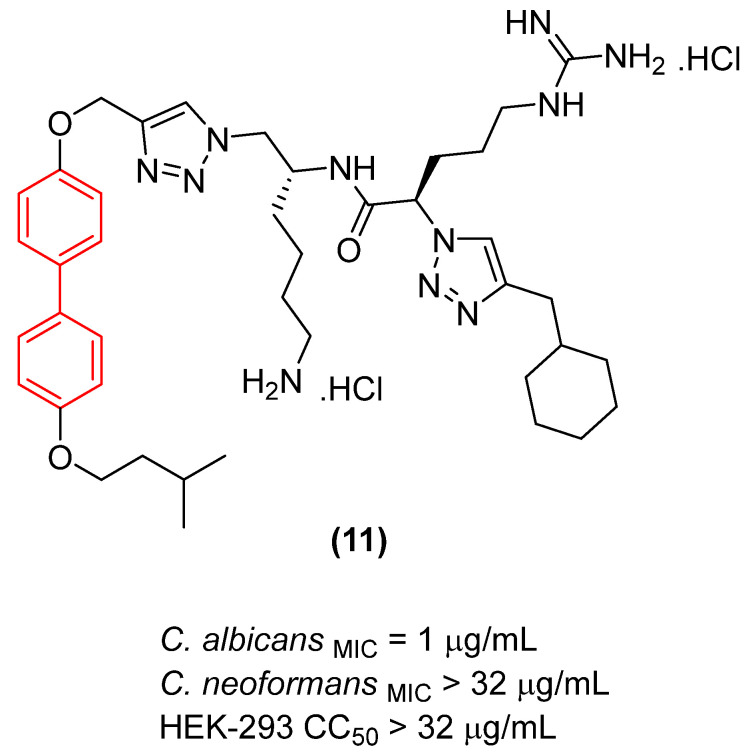
Pharmacological profiling of compound **11**.

Regarding antifungal activity, an evaluation against pathogenic yeasts highlighted the dramatic impact of structural orientation. Compound **11** (Figure 7), the 4,4′-disubstituted isomer, displayed the strongest growth inhibition against *Candida albicans* by a significant margin, achieving an exceptional MIC of 1 μg/mL. The importance of this specific spatial vector is underscored when comparing it to other positional isomers, such as isomers 1, 2, and 3, which displayed substantially weaker efficacy (MIC ≥ 32 μg/mL) against the same pathogen [26].

Interestingly, despite the potent antifungal activity demonstrated by the 4,4′-isomer against *C. albicans*, all tested isomers remained inactive against *Cryptococcus neoformans* var. *grubii* at the tested concentrations. In addition, it showed low cytotoxicity against human embryonic kidney cells, showing significant selectivity. Consequently, compound **11** emerged as a potential selective antifungal agent for *C. albicans*. These findings clearly illustrate that the structural arrangement dictated by the biphenyl core—specifically the 4,4′-substitution pattern—creates an optimal amphiphilic topology that not only enhances antimicrobial efficacy but also drives the specific spectrum of action [26].

**Figure 8:**
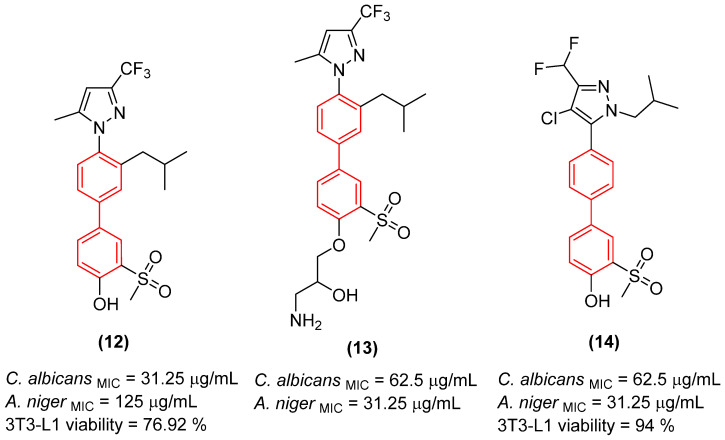
Pharmacological profiling of compounds **12**–**14**.

Furthermore, hybridizing the biphenyl core with nitrogen-rich heterocycles other than standard triazoles has yielded promising results against resistant fungal strains. Walunj et al. [27] reported the synthesis of two series of clubbed 1,1′-biphenyl-pyrazole derivatives designed to target the ergosterol biosynthesis pathway. The unique spatial arrangement provided by the rigid inter-annular biphenyl bond allowed the pyrazole moiety to effectively project its nitrogen atoms for optimal coordination with the heme iron in the CYP51 active site. Detailed structure-activity relationship (SAR) analyses revealed that the electronic and steric nature of the substituents on the biphenyl core severely impacts the antifungal spectrum. Specifically, derivatization at the 4′-position of the biphenyl ring with an alkoxy amino-alcohol group (e.g., compound **13**, Figure 8) or maintaining a 4′-hydroxy group alongside a 4-chloro-3-(difluoromethyl) substituted pyrazole (e.g., compound **14**, Figure 8) generated highly potent agents. These optimized architectures exhibited robust antifungal activity against *Candida albicans* and *Aspergillus niger*, presenting minimum inhibitory concentrations (MICs) between 31.25 and 62.5 µg/mL, which demonstrated efficacy comparable to the clinical standard ravuconazole against *A. niger* [27].

Crucially, the in vitro cytotoxicity evaluation against mammalian cells (mouse embryonic fibroblasts, 3T3-L1) emphasized the necessity of precise structural tuning to achieve a favorable safety profile. While certain highly substituted derivatives displayed significant toxicity with cell viability falling below 50%, the lead antifungal candidates, including compounds **14** and **12** (Figure 8), exhibited no or exceptionally low cytotoxicity, maintaining high cell viability at a concentration of 25 µg/mL. This selective toxicity highlights that the biphenyl-pyrazole scaffold can be rationally optimized to disrupt fungal ergosterol biosynthesis while fully preserving mammalian cell integrity [27].

A summary of these recent SAR insights is provided in Table 3.

#### Cross-Sectional SAR and Recent In Vivo Advances in Antifungal Biphenyls

A critical analysis of the literature involving optimizations and recent in vivo breakthroughs reveals distinct Structure-Activity Relationship (SAR) trends that demonstrate how the biphenyl core is utilized to override classical fungal resistance mechanisms [28,29,30,31]. Table 4 summarizes the most important trends for the SAR of Antifungal Biphenyls with in vivo data.

The primary SAR trend is the application of the unmodified biphenyl core as a highly lipophilic anchor to enhance membrane interaction and target engagement (e.g., CYP51). As demonstrated by Zhao et al., simple imidazole-biphenyl derivatives (Compound **1**) suffered from a lack of broad-spectrum activity [18]. Through structure-guided optimization targeting the CYP51 enzyme, the incorporation of ortho-fluorine atoms onto the biphenyl core (Compound **1m**) induced a conformational twist that significantly enhanced binding, rendering the compound superior to fluconazole against *C. albicans*. However, highly lipophilic biphenyls are susceptible to rapid hepatic metabolism. To overcome this, scaffold hopping strategies are employed. Replacing the imidazole with a triazole ring, combined with the incorporation of electro-reductive groups on the biphenyl rings (Compound **5**), successfully shielded the scaffold from CYP-mediated oxidation, extending the in vitro half-life from 3.6 min to over 145 min [22].

**Figure 9:**
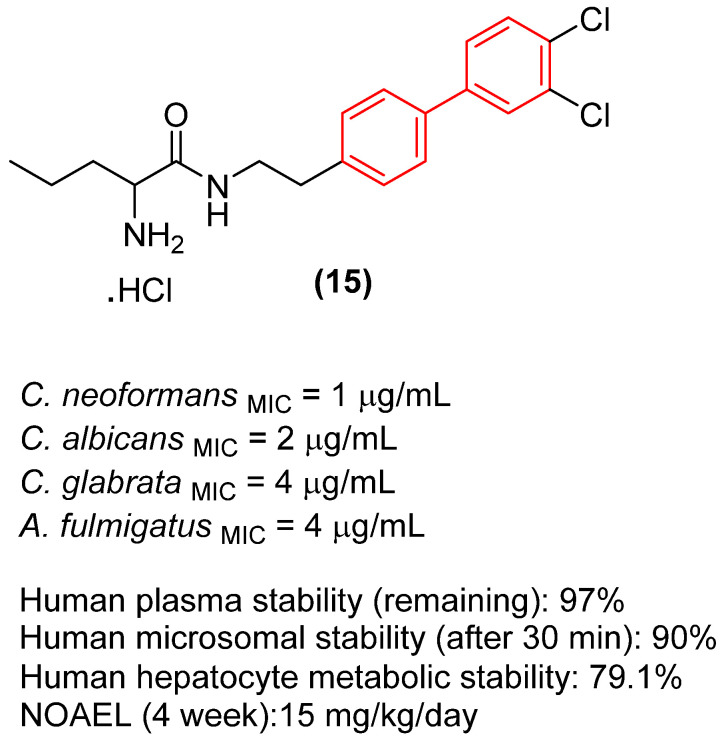
Pharmacological profiling of compound **15**.

New developments have successfully bridged this optimized in vitro potency with in vivo efficacy. Choi et al. demonstrated that rationally designed biphenyl pentanamides, relying on terminal meta/para dichlorination of the biphenyl core, increase lipophilic penetration of the fungal cell wall while simultaneously preventing rapid metabolic degradation. The biphenyl derivative **15** (Figure 9) exhibited extremely rapid fungicidal activity and good in vitro ADME (Figure 9) and, crucially, demonstrated highly potent in vivo efficacy in stringent subcutaneous murine infection models against *Candida* species [31]. The in vivo success of this derivative validates the capability of the optimized biphenyl scaffold to achieve therapeutic tissue concentrations. Notably, while understated in the original report, the substance exhibited moderate toxicity, with cell viability maintained solely at concentrations up to 12.5 μM. Additionally, subacute toxicological evaluations determined the no-observed-adverse-effect level (NOAEL) for compound **15** to be only 15 mg/kg/day over a 4-week exposure period.

### 2.2. Antiviral Activity

#### 2.2.1. Reverse Transcriptase Enzyme Inhibitors

Acquired immunodeficiency syndrome (AIDS), caused by the human immunodeficiency virus (HIV), is currently one of the most prevalent diseases affecting people worldwide. Among the therapeutic targets, the reverse transcriptase (RT) enzyme represents a promising candidate for the development of new anti-HIV substances. Non-nucleoside reverse transcriptase inhibitors (NNRTIs) constitute a significant category of pharmaceutical agents employed in the management of HIV infection. However, the emergence of resistance to the drugs currently in use is a cause for concern and provides justification for the search for new active substances [32,33].

**Figure 10:**
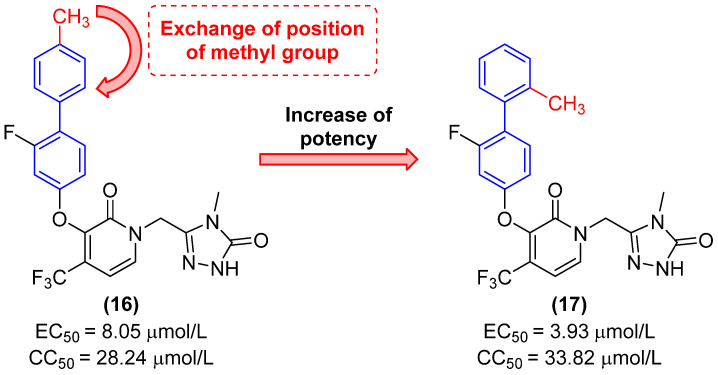
Design of new biphenyl derivatives with activity against HIV.

In this context, Zhao and colleagues [34] synthesized a series of novel biphenyl derivatives with activity against HIV. It was observed that the simple transfer of the methyl from position 4 of the terminal ring of the biphenyl subunit (compound **16**) to position 2 (compound **17**) (Figure 10) resulted in an approximately 2-fold increase in potency against HIV, with derivative **17** exhibiting an EC_50_ value of 3.93 µmol/L. Moreover, a slight reduction in cytotoxicity was observed in comparison to **16** (CC_50_ = 33.82 µmol/L). Molecular docking studies demonstrated that this modification was favorable, widening the dihedral angle of the biphenyl ring and enhancing the interaction between the molecule and the target (Figure 10).

Continuing the series of molecules, Zhao and colleagues [34] synthesized 17 new derivatives with different substituents on both biphenyl rings. All derivatives were assessed for antiviral activity and cytotoxicity in MT-4 cells infected with the WT HIV-1 strain (IIIB). The drug doravirine was employed as a reference point.

**Figure 11:**
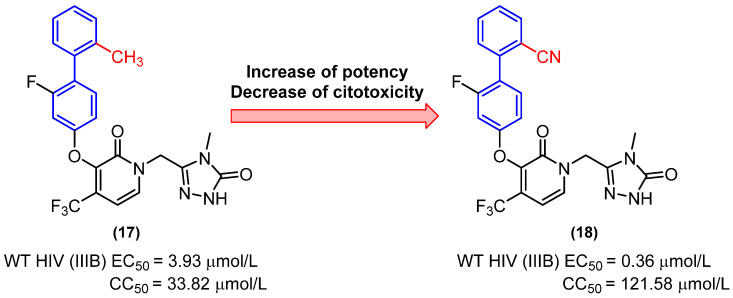
Design of new biphenyl derivatives with activity against HIV.

Derivative **18**, which has a cyano group in place of the methyl group, exhibited a notable potency, with an EC_50_ value of 0.36 µmol/L, approximately 11 times more potent than derivative **17** (EC_50_ = 3.93 µmol/L). Prior in silico studies had indicated that replacing the methyl group with a cyano group would be advantageous, reducing the cytotoxicity of the derivative. This was corroborated by the results of the cytotoxicity evaluation, in which derivative **18** exhibited a CC_50_ of 121.58 µmol/L, approximately threefold less cytotoxic than derivative **17** (CC_50_ = 33.82 µmol/L) in the evaluated cells (Figure 11) [34].

**Figure 12:**
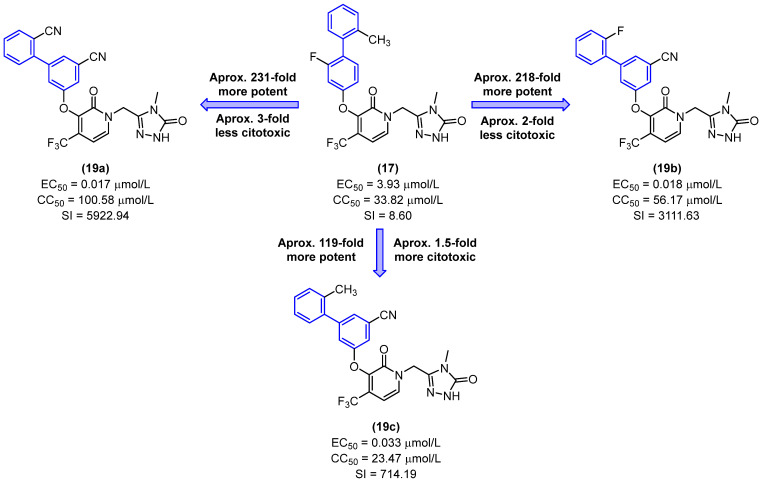
Design of new biphenyl derivatives with activity against HIV.

Replacing the para-biphenyl group of **16**–**18** with the meta-biphenyl group resulted in a notable enhancement in potency, as evidenced by the EC_50_ values of derivatives **19a–c**, which fell within the nanomolar range (Figure 12). Derivatives **19a** and **19b** exhibited potency comparable to that of doravirin, with EC_50_ values of 0.017 and 0.018 µmol/L, respectively. In comparison to the lead compound **17**, derivatives **19a** and **19b** exhibited approximately 231- and 218-fold increases in potency, respectively. Additionally, **19a** and **19b** demonstrated a 3-fold and 2-fold reduction in cytotoxicity relative to **17**, as illustrated in Figure 12 [34].

Considering the encouraging potency demonstrated by the derivatives, the inhibitory activity against mutant strains (L100I, K103N, Y181C, Y188L, E138K, F227L + V106A, and K103N + Y181C) was subsequently assessed. Compounds **19a** and **19b** exhibited greater potency than Doravirin against the K103N and Y188L strains, whereas derivative 9c demonstrated equipotency against the K103N strain (Table 5). Moreover, the compounds demonstrated inhibitory activity at the nanomolar level against the HIV reverse transcriptase enzyme (Table 3) [34].

Yang and colleagues [35] developed a further series of derivatives containing the biphenyl group, which acts as inhibitors of the reverse transcriptase enzyme. In this series, diarylpyrimidine derivatives demonstrated potent activity against HIV-1 (WT) and mutant strains through the fusion of biphenyl and oxime groups. The MTT method was employed to evaluate fourteen new derivatives in MT-4 cells infected with the HIV-1 virus. The drugs nevirapine, efavirenz, and etravirine were utilized as a point of comparison.

**Figure 13:**
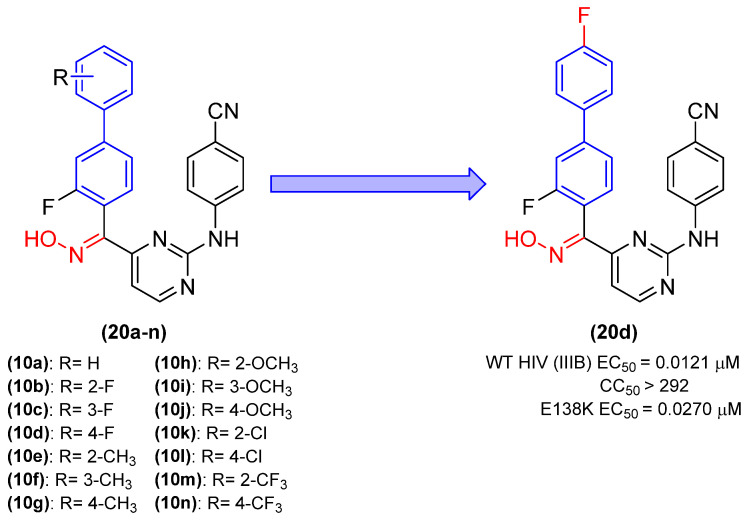
Design of new biphenyl derivatives with activity against HIV.

The results were promising, with inhibitory activity values against HIV-1 between 0.121 and 0.369 µM and a selectivity index (SI) ranging from 71 to 24,105. Derivative **20d**, in which R was replaced by a 4-F group (Figure 13), was the most promising of the series, exhibiting an EC_50_ of 0.121 µM, a lower CC_50_ of >292 µM, and an improved selectivity index of 24,105 [35].

Subsequently, the inhibitory activity of the compounds was assessed against the E138K mutant strain. In accordance with the preceding results, the derivatives demonstrated EC_50_ values spanning a range from 0.027 µM to 1.08 µM. The ortho- and para-substituted derivatives demonstrated the most promising activity, with derivative **20d** once again exhibiting the highest potency against the E138K mutant strain [35]. In the enzymatic evaluation of reverse transcriptase, derivative **20d** exhibited the highest potency, with an IC_50_ value of 0.0421 µM. This was approximately 10 times more potent than the reference drug nevirapine (IC_50_ = 0.450 µM). The findings demonstrate that derivatives comprising biphenyl and oxime groups exhibit antiviral efficacy while displaying minimal cytotoxicity [35].

Recent breakthroughs in the optimization of Non-Nucleoside Reverse Transcriptase Inhibitors (NNRTIs) have further underscored the thermodynamic importance of the biphenyl core when coupled with rationally engineered linkers [36].

**Figure 14:**
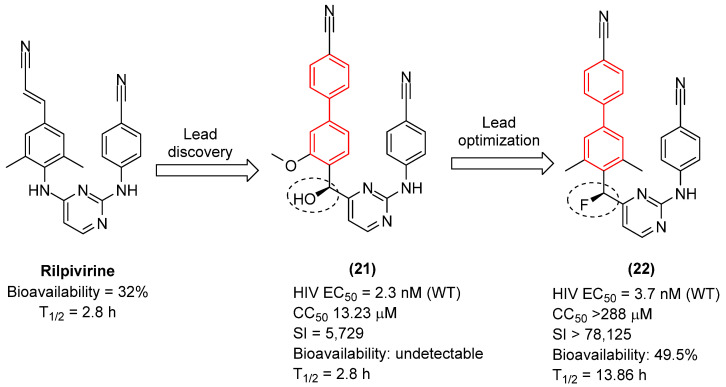
Pharmacological profiling of compounds **21** and **22**.

A recent and prominent study reported the continuation of a research line in which the incorporation of a biphenyl moiety into the diarylpyrimidine (DAPY) pharmacophore—present in the antivirals etravirine and rilpivirine—led to enhanced antiviral activity against various strains. However, the pharmacokinetic profile of the lead compound (**21**, Figure 14) was suboptimal, exhibiting poor metabolic stability. Consequently, a subsequent study by the same group demonstrated that modifying the linker connecting the biphenyl group to the diarylpyrimidine (DAPY) scaffold via targeted halogenation (e.g., forming halomethylene-linked biphenyl-DAPYs) yields exceptional antiviral and pharmacokinetic profiles [36]. The incorporation of halogen atoms (such as fluorine or chlorine) at the methylene bridge restricts the torsional flexibility, effectively locking the molecule into the optimal “horseshoe” conformation required for high-affinity binding within the Non-Nucleoside Inhibitor Binding Pocket (NNIBP) [36]. Moreover, the halomethylene linker serves as a crucial bioisostere that sterically and electronically masks metabolically vulnerable hydroxyl sites, thereby preventing rapid Phase I oxidation and glucuronidation [36]. The optimized (S)-enantiomers of these fluoromethylene-biphenyl derivatives (**22**, Figure 14) exhibited remarkable sub-nanomolar potency against wild-type and single-mutant HIV-1 strains, showcasing a significantly improved selectivity index (SI > 78,125) and extended in vivo oral bioavailability and half-life (T_1/2_) [36].

#### 2.2.2. HIV Protease Enzyme Inhibitors

**Figure 15:**
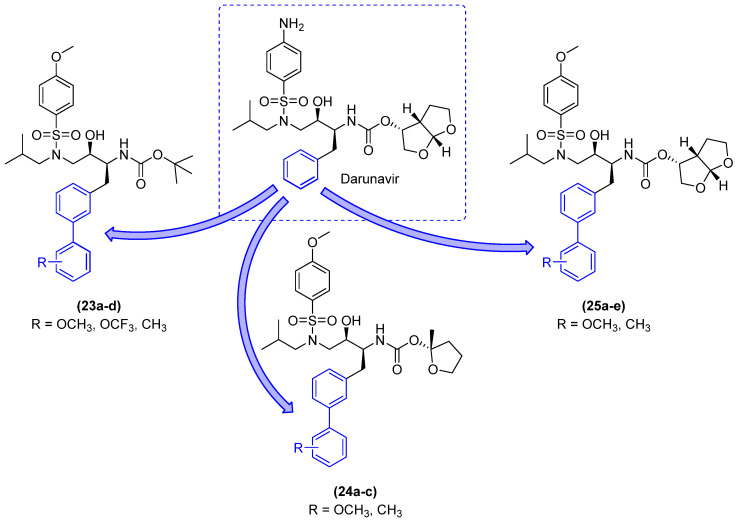
Design of new biphenyl derivatives with activity against HIV protease.

HIV protease inhibitors are a cornerstone of antiretroviral treatment. However, the emergence of strains resistant to these drugs has prompted significant concerns about the future of HIV treatment using this pharmacological approach. In response to this concern, Gosh and colleagues [37] developed a series of HIV-1 protease inhibitors with the objective of achieving activity against multidrug-resistant variants. The project was based on the superposition of the X-ray structures of the HIV-1 protease in complex with the drugs darunavir and saquinavir. The authors explored the potential of incorporating substituents into the benzyl ring of Darunavir to enhance hydrophobic interactions with the HIV-1 protease, as well as incorporating hydrophobic groups to augment the lipophilicity of these derivatives. Derivatives **23a–d**, **24a–c**, and **25a–e** feature a substituted biphenyl in lieu of the benzyl ring (Figure 15). This is attributable to the high degree of selectivity (CC_50_ > 292 µM) and superior selectivity index (SI = 24105) [37].

The derivatives were evaluated in an enzyme inhibition assay and subsequently subjected to antiviral evaluation. Derivative **23a** demonstrated the most potent enzyme inhibition, with a Ki value of 0.82 nM. However, in the antiviral evaluation, it exhibited an IC_50_ greater than 1 µM. The remaining derivatives in the **23a–d** series demonstrated low potency in the enzymatic assay and no notable antiviral activity (Table 6). In the **24a–c** series, there was a notable enhancement in both enzyme inhibition and antiviral activity. Derivative **24a**, for instance, exhibited a Ki value of 0.58 nM and an IC_50_ of 0.18 µM (Table 6), which is indicative of a promising outcome. The 13a–e series demonstrated the most promising results of the three. The derivatives exhibited potent enzyme inhibition and antiviral activity. Derivative **25e** exhibited potent inhibitory activity with a Ki value of 0.012 nM and antiviral activity with an IC_50_ value of 0.003 µM, as illustrated in Table 6 [37].

Based on these findings, derivative **25e** was chosen for further analysis using a panel of multidrug-resistant HIV-1 variants, with darunavir and aprenavir serving as controls (see Table 7). Derivative **25e** demonstrated remarkable potency against all multidrug-resistant HIV-1 variants tested, with EC_50_ values ranging from 0.0029 to 0.036 µM [37].

#### 2.2.3. Coronavirus Disease 2019 (COVID-19)

The Coronavirus Disease 2019 (COVID-19) pandemic has had a profound impact on the global economy and public health, resulting in the deaths of thousands of individuals worldwide. The Mpro enzyme, also designated 3CLpro, is the primary protease of SARS-CoV-2, the virus that causes COVID-19. It is responsible for cleaving polyproteins into multiple functional proteins. Given the fundamental role of this protease in viral replication, Mpro has emerged as a promising target for the development of new drugs in the fight against SARS-CoV-2, the virus that causes the disease known as “coronavirus disease 2019” (COVID-19) [38,39].

The compound ML188 (R) is a non-covalent inhibitor of SARS-CoV Mpro, developed through HTS (High-throughput Screening), with an IC_50_ value of 1.5 ± 0.3 µM in terms of Mpro inhibition and in vitro antiviral activity in Vero E6 cells with an EC_50_ value of 12.9 µM [40]. Utilizing this compound as a template, Kitamura and colleagues [41] devised 39 variants as prospective inhibitors of SARS-CoV-2 Mpro, aiming to identify not only enzymatic activity but also potent antiviral activity.

**Figure 16:**
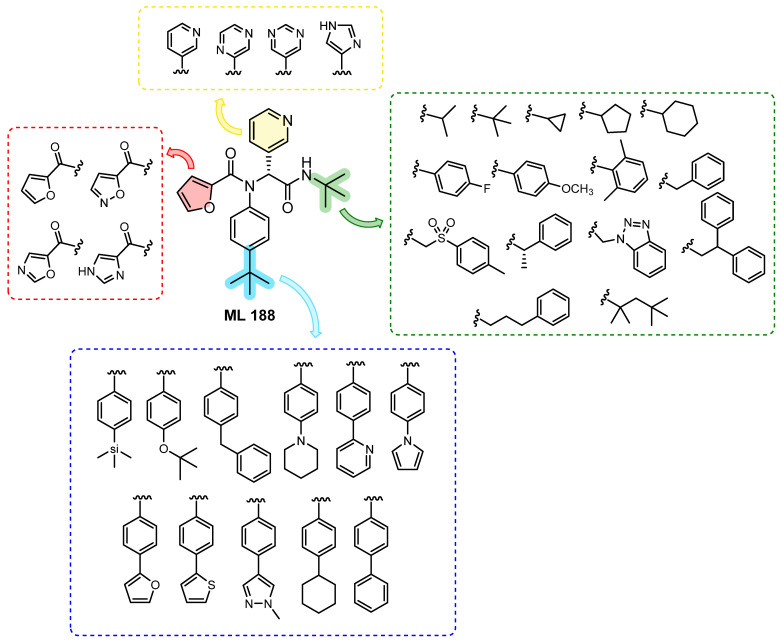
Design of novel non-covalent SARS-CoV-2 Mpro inhibitors.

Among the structural modifications proposed through the superposition of compound ML188 with the structure of SARS-CoV (PDB: 3V3M), it was observed that substitutions in the tert-butyl groups (in green and blue) by aromatic rings or heterocycles could favor the interaction with the enzyme, resulting in an increase in the biological activity of these compounds (Figure 16). Modifications to the furan and pyridine rings were proposed in the design of these derivatives, as these functional groups form important hydrogen bonds with Mpro [41].

**Figure 17:**
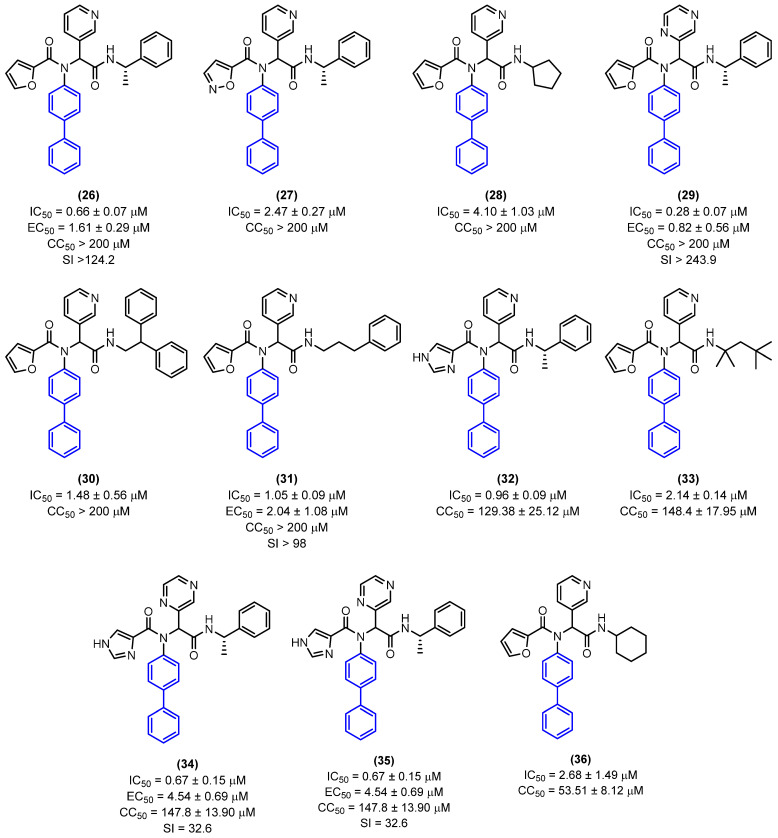
Design of novel non-covalent SARS-CoV-2 Mpro inhibitors.

In the initial stage of the study, all compounds were evaluated in the enzymatic assay as mixtures of enantiomers or diastereoisomers. The IC_50_ values were determined for the derivatives that exhibited more than 50% inhibition at 20 µM. Several derivatives within the series exhibited a notable enhancement in enzyme inhibition relative to the prototype ML188 (R), with IC_50_ values lower than 3 µM. Following the determination of the IC_50_, derivatives with IC_50_ values lower than 5 µM were selected for a cytotoxicity test in Vero E6 cells, a cell line utilized in the antiviral evaluation against SARS-CoV-2. This was done with the objective of prioritizing compounds for in vitro antiviral activity. The biphenyl derivatives evaluated exhibited low cytotoxicity. Among them, **26**, **27**, **28**, **29**, **30**, and **31** demonstrated CC_50_ values exceeding 200 µM, while derivatives **32**, **33**, and **34** exhibited CC_50_ values of 129.38 ± 25.12 µM, 148.4 ± 17.95 µM, and 147.8 ± 13.90 µM, respectively. Derivatives **35** and **36** demonstrated cytotoxicity below 100 µM, with CC_50_ values of 67.05 µM and 53.51 µM, respectively (Figure 17). To perform in vitro antiviral activity assays with SARS-CoV-2 in Vero E6 cells, the derivatives that demonstrated enzymatic inhibition with an IC_50_ value of less than 1 μM and a CC_50_ value greater than 100 μM were selected. The compound ML188 was employed as a control. The derivatives with the biphenyl subunits **26**, **29**, **31**, and **34** exhibited potent antiviral activity with EC_50_ values ranging from 0.82 μM to 4.54 μM, exhibiting greater potency than ML188 (EC_50_ > 20 μM) (Figure 17) [41].

**Figure 18:**
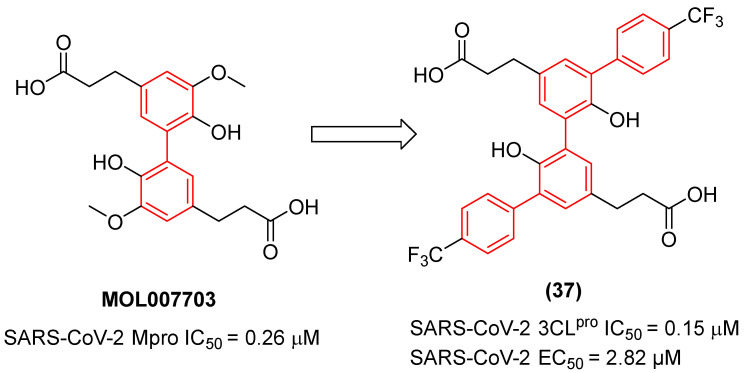
Pharmacological profiling of compound **37**.

Furthermore, the therapeutic scope of the biphenyl scaffold against SARS-CoV-2 has recently been expanded through the discovery of dual-activity inhibitors [42]. Shang et al. identified a natural biphenyl-containing product, MOL007703 (Figure 18), extracted from *Stellariae radix*, as a promising structural starting point for targeting the highly conserved main protease (Mpro or 3CLpro) of SARS-CoV-2 [42]. Through systematic structure-based optimization, the researchers executed a sophisticated structure-activity relationship (SAR) exploration that significantly expanded the hydrophobic topology of the lead. A pivotal modification was the introduction of an additional phenyl ring bearing a para-trifluoromethyl group, which effectively doubled the biphenyl character of the resulting optimized derivative, compound **37** (Figure 18). This strategic duplication of the biphenyl motif proved thermodynamically critical: the newly introduced fluorinated phenyl ring acts as a massive hydrophobic extension that deeply penetrates and optimally fills the highly lipophilic S4 pocket of the 3CLpro enzyme. Simultaneously, the strong electron-withdrawing effect of the trifluoromethyl group introduces favorable electrostatic and dipole interactions, acting synergistically with the core biphenyl to maximize the hydrogen-bonding network—particularly with key catalytic residues in the polar S1 pocket—and overall van der Waals contacts. As a direct consequence of this expanded bis-biphenyl-like architecture, compound **37** showed superior 3CLpro inhibitory activity with an IC_50_ of 0.15 µM, proving 13.6-fold more potent than the reference covalent inhibitor ebselen (IC_50_ = 2.04 µM) and dramatically outperforming the original natural product MOL007703. This robust structural engagement translates directly to potent cellular antiviral efficacy (EC_50_ = 2.82 µM), which is highly comparable to that of remdesivir. Notably, this optimized biphenyl series also exhibited significant dual-functional bioactivity by suppressing bladder cancer cell lines (T24), demonstrating the exceptional polypharmacological potential of expanding the strict biphenyl core to target complex, concurrent pathological networks without sacrificing specific antiviral affinity [42].

#### 2.2.4. Host-Targeting Hepatitis B Virus (HBV) Agents: p38 MAPK Inhibition

**Figure 19:**
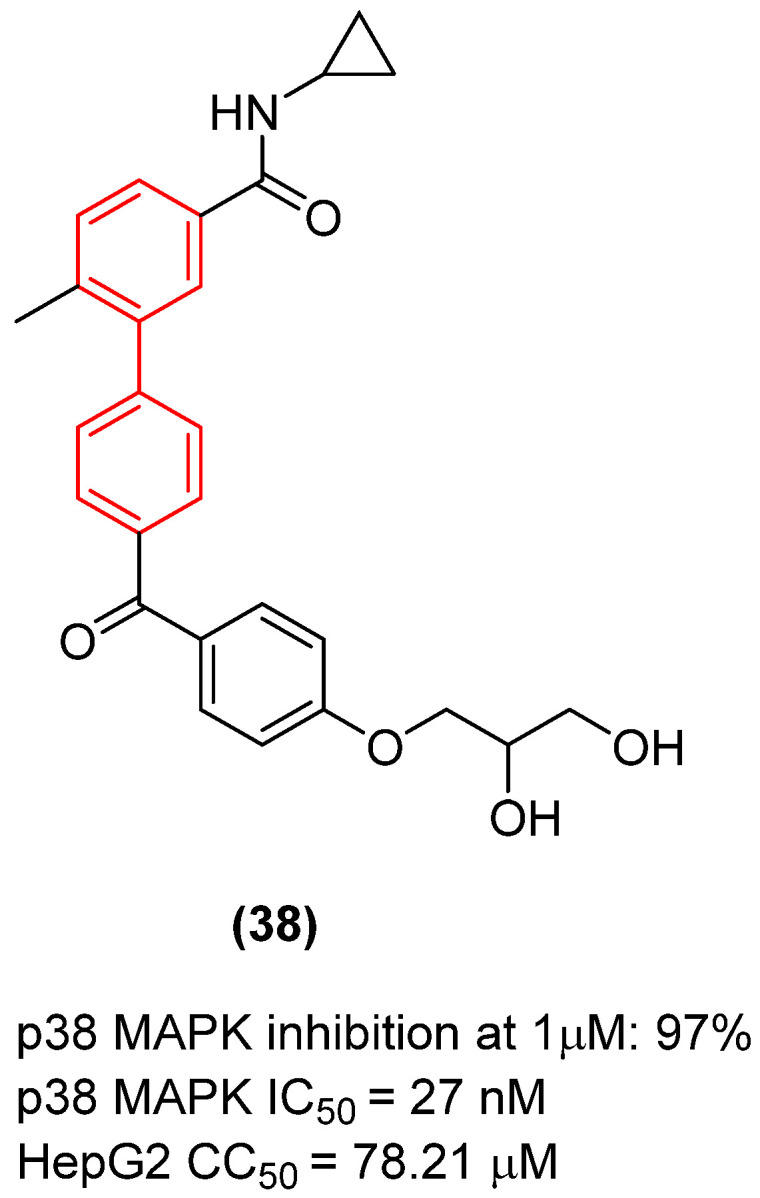
Pharmacological profiling of compound **38**.

In a profound paradigm shift from targeting mutable viral proteins, the biphenyl scaffold has been successfully employed to target conserved host cell factors hijacked during viral replication [43]. A striking example of this host-directed antiviral therapy is compound **38** (Figure 19), a novel and highly selective biphenyl-based p38 mitogen-activated protein kinase (MAPK) inhibitor with important activity against Hepatitis B virus (HBV). Kim et al. demonstrated that **38** exhibits potent antiviral activity against HBV, with moderately low cytotoxicity to a panel of mammalian cells, including hepatocarcinoma cells (HepG2), a host cell of HBV. Rather than directly inhibiting the viral polymerase, the biphenyl derivative suppresses HBsAg secretion and viral DNA synthesis by blocking the virus-induced activation of the host p38 MAPK signaling pathway. The strict biphenyl core of **38** is structurally indispensable; it wedges perfectly into the hydrophobic allosteric pocket of the host kinase, ensuring high selectivity over other cellular kinases and preventing cytotoxicity. Because it targets a stable host protein rather than a rapidly mutating viral target, this biphenyl-driven mechanism offers a high barrier to viral resistance, positioning the biphenyl scaffold as a privileged structure for robust, host-directed antiviral therapeutics [43].

#### 2.2.5. Cross-Sectional SAR, Conformational Locking, and In Vivo Efficacy in Antiviral Agents

The pharmacological versatility of the strict biphenyl core in antiviral drug discovery is intrinsically linked to its unique thermodynamic and conformational properties. As summarized in Table 8, the rotational freedom around the central C–C bond allows the biphenyl system to adopt multiple dihedral angles, oscillating between planar and twisted conformations depending on the steric hindrance of ortho-substituents. This conformational adaptability enables the biphenyl motif to act as a privileged structural anchor across a diverse array of viral and host targets, maximizing essential van der Waals and π-π interactions within complex hydrophobic pockets.

In the context of HIV-1, ortho-substituted biphenyls effectively lock the dihedral angle to complement the three-dimensional topography of the RT non-nucleoside inhibitor binding pocket (NNIBP) or the mutated S1′ subsite in MDR proteases. Furthermore, recent advances have demonstrated that the biphenyl scaffold can be rationally engineered to overcome traditional pharmacokinetic liabilities. For instance, the halogenation of linkers attached to the biphenyl core (e.g., halomethylene-biphenyl-DAPYs) restricts torsional flexibility into an optimal “horseshoe” shape while simultaneously bioisosterically shielding metabolically vulnerable sites from rapid Phase I oxidation and glucuronidation, drastically increasing in vivo half-life and oral bioavailability [36].

Beyond HIV, the core has driven breakthroughs in combating emerging and resilient viral pathogens. In SARS-CoV-2 therapeutics, expanding the hydrophobic topology by doubling the biphenyl character (as seen in the structural evolution from the natural product MOL007703 to compound **37**) allows the extended bis-biphenyl-like architecture to deeply penetrate and perfectly fill the highly lipophilic S4 pocket of the 3CLpro enzyme [42]. This strategic topological expansion maximizes thermodynamic stabilization, providing highly potent, dual-functional efficacy against viral replication and bladder cancer cell proliferation [42].

Moreover, the therapeutic scope of biphenyls extends significantly into broad-spectrum and host-directed antiviral strategies. Most innovatively, the biphenyl motif enables host-directed therapies by selectively wedging into the allosteric hydrophobic pockets of conserved host kinases. A prime example is the biphenyl-based p38 MAPK inhibitor **38**, which suppresses HBV replication by blocking virus-induced host signaling [42]. By targeting a stable host factor rather than a mutable viral protein, this biphenyl-driven mechanism offers a high barrier to resistance, circumventing classical viral mutation pathways entirely [42].

Collectively, these cross-sectional SAR trends—ranging from dynamic conformational locking and metabolic shielding to topological expansion and precise host-kinase targeting—validate the strict biphenyl scaffold not merely as a passive lipophilic spacer, but as an active, thermodynamic driver of broad-spectrum antiviral efficacy and in vivo safety.

### 2.3. Antibacterial Activity

The introduction of antibiotics has been regarded as a significant medical advancement since the 20th century, as they have become a mainstay in the treatment of various bacterial infections. However, the indiscriminate and irrational use of antibiotics, in addition to the gradual decline in the discovery and development of these drugs and the increase in bacterial resistance, represents a significant threat to public health, undermining the benefits of this therapeutic approach [44,45].

The 2022 Global Antimicrobial Resistance and Surveillance System (GLASS) report indicates that there are alarming rates of bacterial resistance, which presents a significant challenge to the effective treatment of these infections. The prevalence of third-generation cephalosporin-resistant *Escherichia coli* and methicillin-resistant *Staphylococcus aureus* in 76 countries is a matter of concern. Furthermore, *Klebsiella pneumoniae* has also demonstrated high levels of antibiotic resistance, which is a significant global concern. As the effectiveness of these drugs becomes compromised, the risk of untreatable infections increases [46].

**Figure 20:**
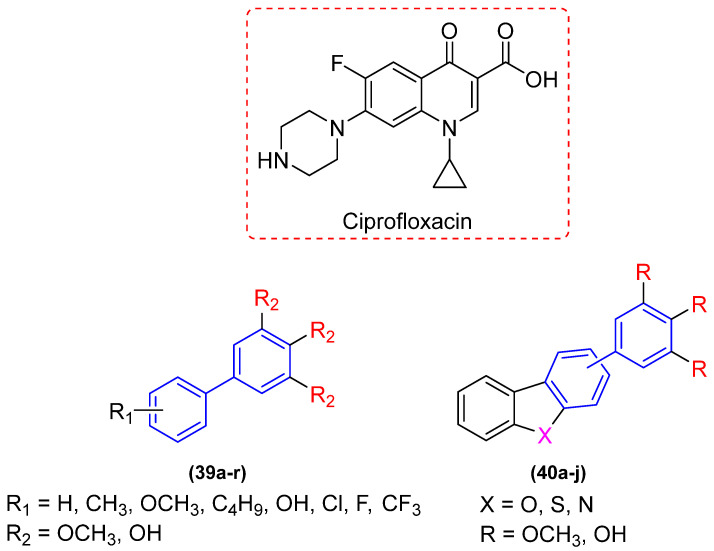
New derivatives of biphenyl phytoalexins and benzoheterocycles as potential antimicrobial agents.

In an effort to alter this situation, Wang and colleagues [47] devised two collections of novel biphenyl phytoalexin and benzoheterocycle derivatives as prospective antimicrobial agents against antibiotic-resistant bacterial strains (Figure 20). Derivatives **39a–r** and **40a–j** were evaluated for in vitro antimicrobial activity in eight resistant bacterial strains through the broth microdilution test to determine the MIC. Ciprofloxacin was used as a positive control.

**Figure 21:**
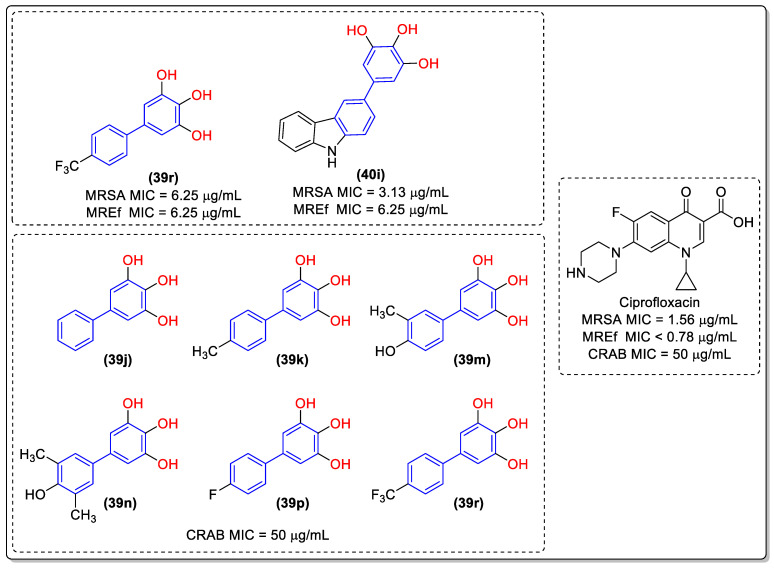
Activity of new biphenyl phytoalexin derivatives and benzoheterocycles.

Of the 28 synthesized compounds, **39a–r** and **40a–j**, 11 demonstrated antibacterial activity against Gram-positive bacteria. The compounds bearing a hydroxyl group in the R_2_ position in **39a–r** and an R in **40a–j** exhibited moderate to significant inhibitory activity, indicating that the presence of a hydroxyl group in the biphenyl ring plays a pivotal role in impeding bacterial growth when compared with the derivatives bearing a methoxyl group, which did not demonstrate any inhibitory effect against resistant strains, presenting MIC values exceeding 100 µg/mL. Derivatives **39r** and **40i** are of particular interest, exhibiting inhibitory activity with MIC values of 6.25 and 3.13 µg/mL, respectively, against MRSA (methicillin-resistant *Staphylococcus aureus*) and MREf (multidrug-resistant *Enterococcus faecalis*) (Figure 21). Moreover, compounds **39j**, **39k**, **39m**, **39n**, **39p**, and **39r** exhibited inhibitory activity against Gram-negative bacteria CRAB (carbapenem-resistant *Acinetobacter baumannii*) with an identical MIC value as that of ciprofloxacin (MIC 50 µg/mL) (Figure 21). The results substantiate the assertion that the biphenyl subunit plays a pivotal role in the advancement of novel drug candidates designed to circumvent bacterial resistance [47].

Tuberculosis (TB) is an infectious disease caused by the bacterium Mycobacterium tuberculosis. It affects more than 10 million people each year and is one of the leading causes of death worldwide. As reported by the World Health Organization, tuberculosis is a global health concern, with higher incidence rates observed in low- and middle-income countries. Additionally, it is the leading cause of mortality among individuals with HIV, accounting for 167,000 deaths in 2022 [48,49].

Multidrug-resistant tuberculosis (MDR-TB) represents a significant public health concern, and the effort to address this multidrug resistance remains a global priority, necessitating the development of highly challenging treatment regimens [50,51,52]. A promising target for overcoming the problem of multidrug resistance to currently available TB treatments is the protein tyrosine phosphatase B of Mycobacterium tuberculosis (MptpB), which is secreted into the host cytoplasm. MptpB is an essential virulence factor, indispensable for the survival of the pathogen. The literature indicates that targeting the virulence factors of the pathogen may be an effective strategy for preventing infection and limiting the survival of the pathogen within the host [51,52,53].

**Figure 22:**
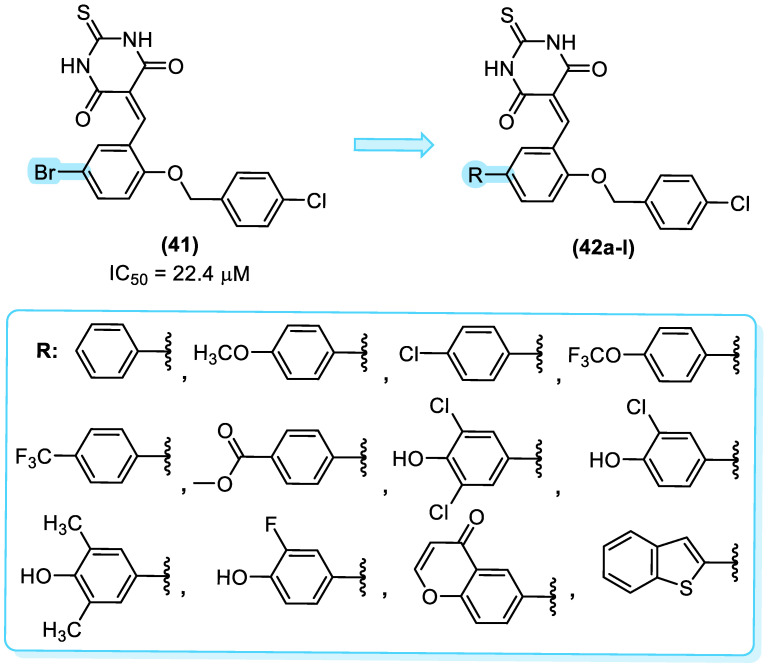
New derivatives against MptpB.

A virtual screening revealed that compound **41** (Figure 22) exhibited modest potency against MptpB, with an IC_50_ value of 22.4 µM. Cheng and colleagues [54] utilized these data as a foundation for the development of novel structure-based MptpB inhibitors (**42a–l**, Figure 22), targeting compounds with enhanced potency and efficacy. Initially, a molecular docking study was conducted, which revealed that the bromine atom could be replaced by aryl substituents to occupy the P1 pocket of MptpB. This indicated that this group would have a superior interaction and, consequently, promote an increase in the inhibition of MptpB. To evaluate the inhibitory activity, several substituents were introduced in the R position (Figure 22) [54].

**Figure 23:**
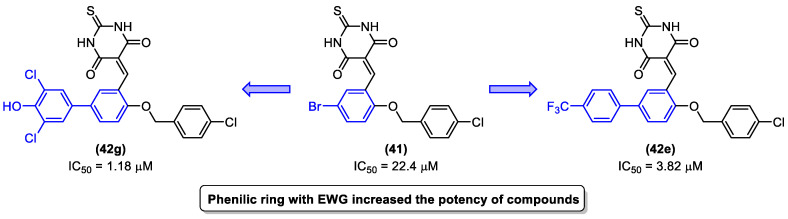
Proposed New Derivatives Against MptpB.

Cheng and colleagues [54] observed that the introduction of a phenyl group with electron-withdrawing groups, such as chlorine and trifluoromethoxy, resulted in enhanced inhibitory activity compared to compound **41**. The compounds bearing 4-trifluorophenyl and dichlorophenol groups exhibited a notable enhancement in activity against MptpB, with IC_50_ values of 3.82 µM and 1.18 µM, respectively (Figure 23).

In addition to MptpB inhibition, the compounds were evaluated for their intracellular anti-TB activity in macrophages and for their cytotoxicity in Vero cells and macrophages (J774A). Isoniazid and rifampicin, the first-line drugs for treating tuberculosis, were employed as controls in the assays. The compounds did not exhibit notable antimycobacterial activity, as evidenced by IC_50_ values exceeding 32 µg/mL. However, they did not demonstrate any evidence of cytotoxicity in Vero cells, with IC_50_ values exceeding 64 µg/mL [54].

Another potential target for the development of new antibacterial agents is FtsZ (Filamenting Temperature-Sensitive Mutant Z), a prokaryotic cell division protein that is the most abundant and highly conserved. Given its pivotal function in bacterial viability, FtsZ has been identified as a promising target for the inhibition of bacterial cytokinesis, which ultimately results in cell lysis [55,56,57].

**Figure 24:**
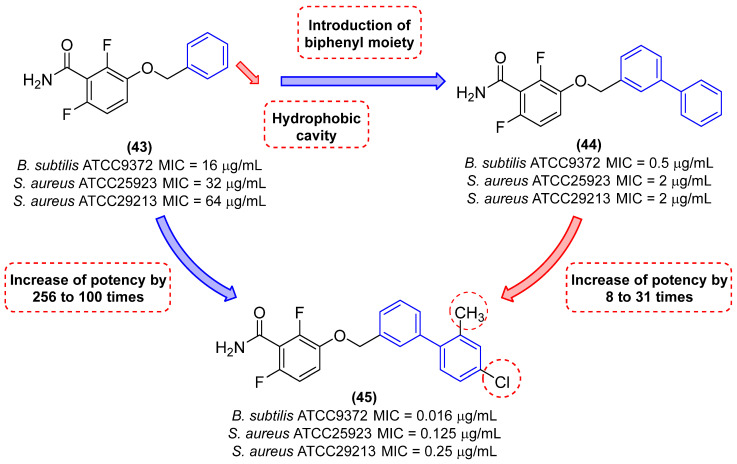
Design of new derivatives with potential bacterial activity.

Deng and colleagues [7] developed a series of biphenyl benzamides with the objective of evaluating antibacterial activity, cytotoxicity, and pharmacokinetics. The results demonstrated that the replacement of the benzene ring with a biphenyl subunit resulted in enhanced activity, with derivative **44** exhibiting approximately 32-, 16-, and 32-fold greater potency than **43** against *B. subtilis* (ATCC9372), *S. aureus* (ATCC25923), and *S. aureus* (ATCC29213), respectively (Figure 16). Compound **45** (Figure 24) exhibited the highest potency within the series, with MIC values of 0.016 µg/mL against *B. subtilis* ATCC9372 and 0.125 µg/mL against *S. aureus* ATCC2592. Derivative **45** demonstrated the greatest potency, with MIC values of 0.125 and 0.25 µg/mL against *S. aureus*. This was 256- to 1000-fold more potent than 29 and approximately 8- to 31-fold more potent than **43** (Figure 24). Moreover, derivative **45** exhibited a potency approximately 62-, 4-, and 4-fold greater than that of vancomycin against *B. subtilis* (ATCC9372), *S. aureus* (ATCC25923), and *S. aureus* (ATCC29213), respectively [7].

**Figure 25:**
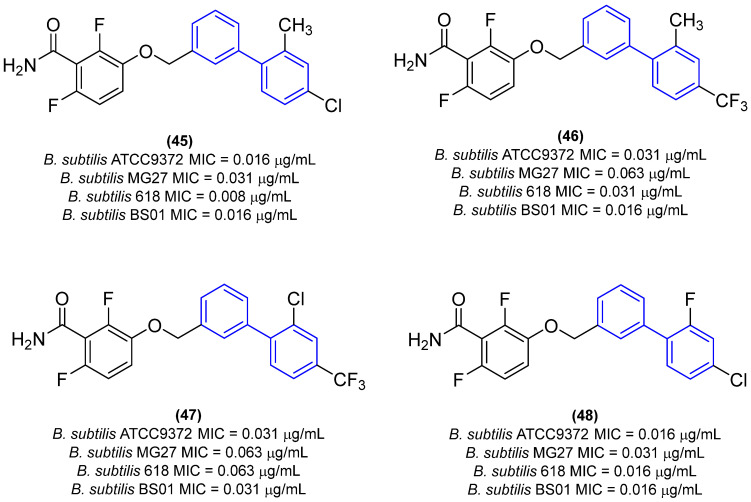
In vitro antibacterial activity of compounds **45–48** against four different strains of *B. subtilis*.

The most potent derivatives against *B. subtilis* ATCC9372 (**45**–**48**, Figure 25) were evaluated against three additional *B. subtilis strains*, demonstrating MIC values ranging from 0.008 µg/mL to 0.063 µg/mL (Figure 25) [7].

Derivative **45** demonstrated the most promising activity, exhibiting an MIC of 0.031 µg/mL against *B. subtilis* MG27, 0.008 µg/mL against *B. subtilis* 618, and 0.016 µg/mL against *B. subtilis* BS01. Subsequently, it was evaluated against a variety of pathogenic bacteria, and no antibacterial activity was observed against Gram-negative bacterial strains [7]. Cytotoxicity results demonstrated that the compounds did not exhibit cytotoxicity in such cell lines (CC_50_ > 20 µg/mL), with favorable selectivity indices (SI > 645), indicating that these compounds are highly selective. Given its potent antibacterial activity and low cytotoxicity, the MBC of compound **45** was evaluated against *S. aureus* ATCC25923 and *B. subtilis* ATCC9372 to ascertain whether the compound was bactericidal or bacteriostatic. In accordance with CLSI protocols, an antibiotic is classified as bactericidal when the MBC/MIC ratio is equal to or less than four, whereas a bacteriostatic antibiotic has an MBC/MIC ratio greater than four. The results demonstrated that compound **45** exhibited bactericidal behavior, as evidenced by an MBC/MIC ratio of 2 against *S. aureus* ATCC25923 and an MBC/MIC ratio of 4 against *B. subtilis* ATCC9372. These findings substantiate the potential of biphenyl benzamides as promising FtsZ inhibitors and antibacterial agents [7].

**Figure 26:**
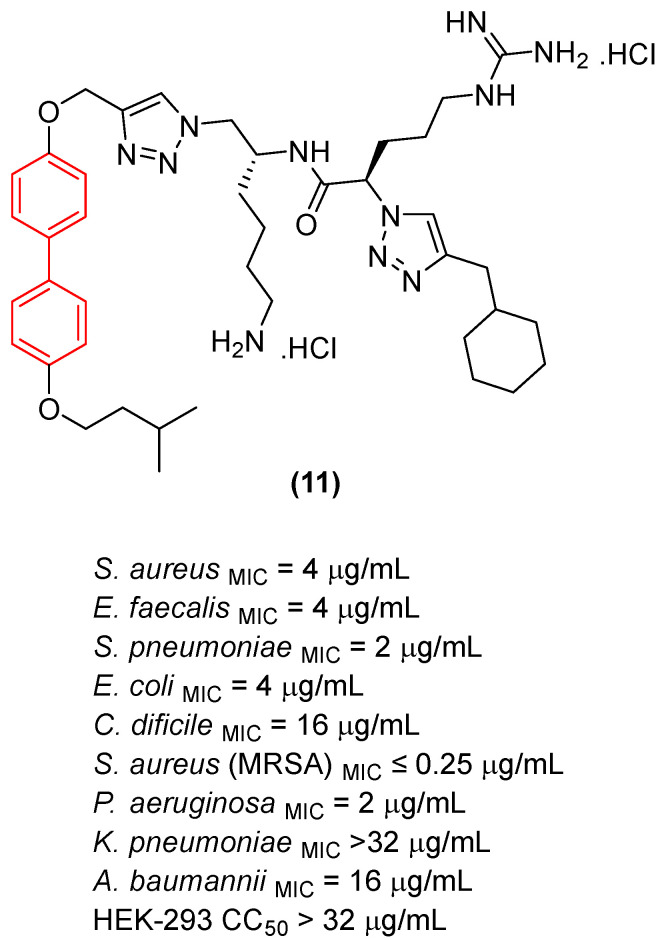
Pharmacological profiling of compound **11**.

In addition to these foundational studies, recent explorations of the biphenyl scaffold have expanded its utility against both Gram-positive and Gram-negative resilient pathogens, demonstrating its versatility across various chemotypes. Tague et al. [26] synthesized a series of small-molecule antimicrobial peptidomimetic amphiphiles (SMAPs) to investigate the role of spatial positioning of the biphenyl core. They demonstrated that positional isomerism profoundly impacts antibacterial efficacy. The substitution of the peptidomimetic chain and the hydrophobic isopentyl group in a 3,2′-arrangement (Compound **11**, Figure 26) resulted in a 4- to 8-fold increase in efficacy against pathogenic Gram-negative bacteria, such as *Pseudomonas aeruginosa* and *Escherichia coli*, achieving a minimum inhibitory concentration (MIC) of 2 µg/mL. In contrast, the baseline 2,2′-isomers were significantly less active, proving that molecular shape and facial amphiphilicity are critical for penetrating the robust Gram-negative outer membrane [26]. Notably, as shown in Section 2.1, in addition to demonstrating significant efficacy against a broad panel of bacteria, Compound **11** exhibited pronounced antifungal activity. These findings highlight the potential of this class of compounds in targeting a wide spectrum of pathogens associated with opportunistic infections [26].

**Figure 27:**
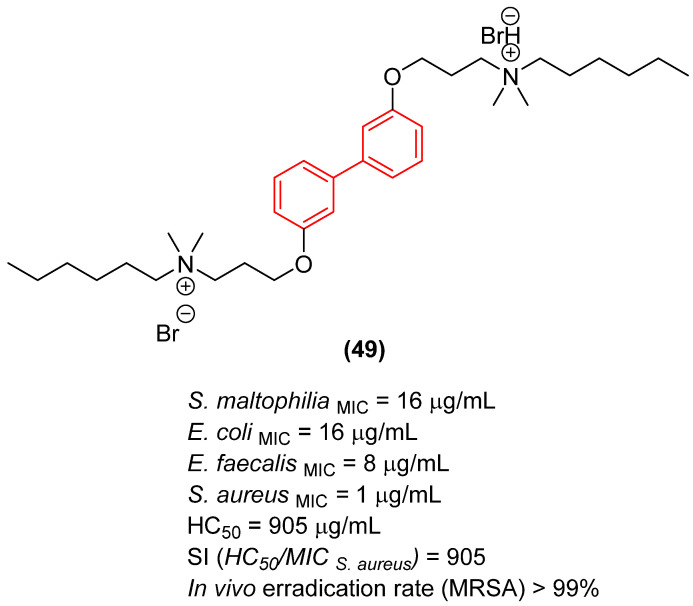
Pharmacological profiling of compound **49**.

Similarly focusing on membrane-active agents, Li et al. [58] utilized the biphenyl core as a rigid linker in the development of biaromatic quaternary ammonium cationic peptidomimetics. The study revealed that the distance between substituents on the biphenyl rings critically influences both antibacterial potency and hemolytic toxicity. The 3,3′-substituted derivative (Compound **49**, Figure 27) exhibited optimal performance, displaying potent bactericidal activity against methicillin-resistant *Staphylococcus aureus* (MRSA) with an MIC of 1 µg/mL. Importantly, this specific biphenyl linkage restricted conformational flexibility in a manner that favored selective interaction with negatively charged phosphatidylglycerol (PG) and cardiolipin (CL) on bacterial membranes, thereby maintaining an exceptionally low hemolytic profile against mammalian red blood cells (HC_50_ = 905 µg/mL) [58]. In vivo studies in mice demonstrated that compound **49** exhibits superior antibacterial activity against MRSA compared to vancomycin. At equivalent concentrations, treatment with the compound reduced the cutaneous bacterial load more significantly, achieving eradication rates exceeding 99% at both 5 and 10 mg/kg/day doses [58].

**Figure 28:**
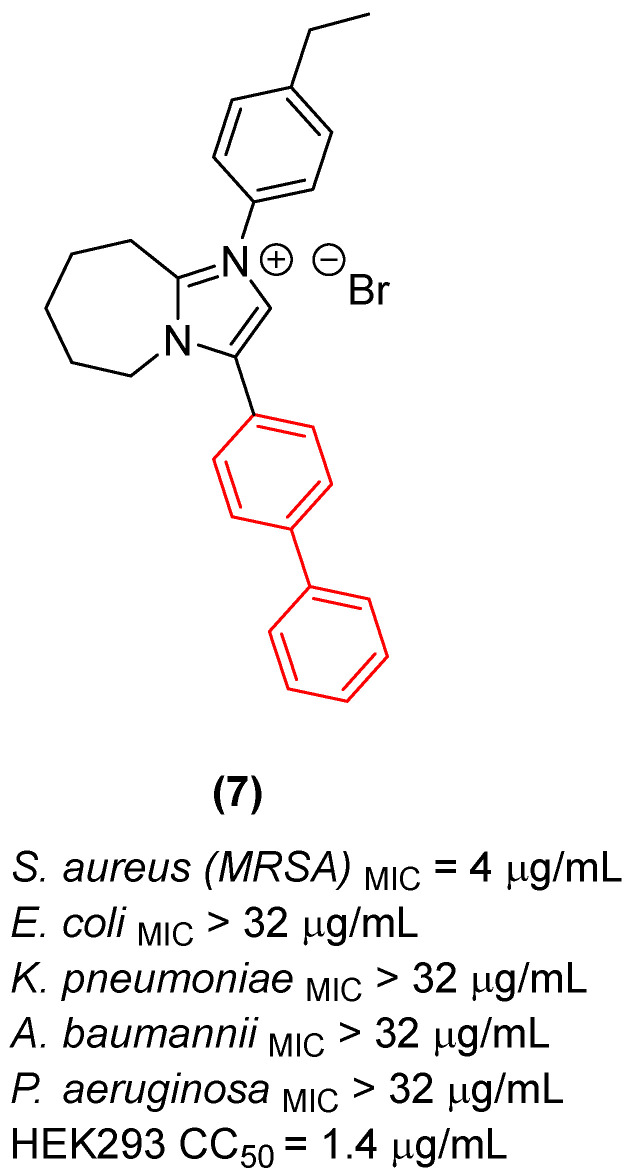
Pharmacological profiling of compound **7**.

Furthermore, the incorporation of the biphenyl moiety into heterocyclic systems has been extensively employed to increase overall lipophilicity and target residency time. Demchenko et al. [24] synthesized a series of 3-biphenyl-3H-imidazo[1,2-a]azepin-1-ium bromides. Their results confirmed that the bulky biphenyl group at position 3 is essential for driving activity against *S. aureus* MRSA, with derivative **7** (Figure 28) exhibiting an MIC of 4 µg/mL. Scaffold hopping from a 7-membered to a 6-membered ring system severely diminished efficacy, establishing that the precise spatial orientation of the biphenyl ring relative to the azepine core is pharmacophorically critical [24]. Nevertheless, the antibacterial activity of this compound class, including **7**, was strictly selective for *S. aureus*, with the entire series showing no growth inhibition of *E. coli*, *K. pneumoniae*, *A. baumannii*, and *P. aeruginosa*. Additionally, while compound **7** demonstrated notable antifungal activity, its high cytotoxicity hampers its selectivity and further preclinical development [24].

**Figure 29:**
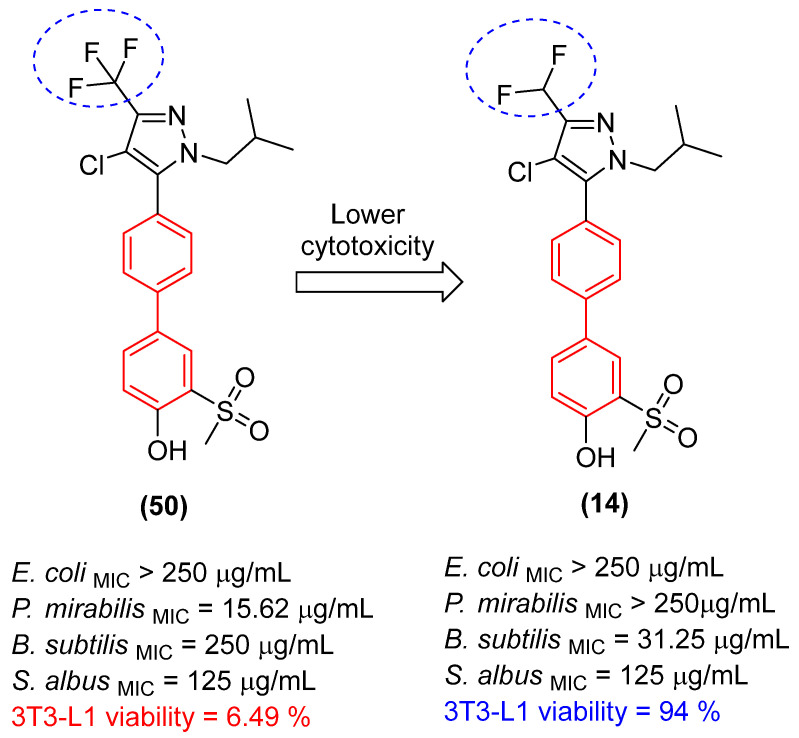
Pharmacological profiling of compound **50** and **14**.

Likewise, Walunj et al. [27] demonstrated the profound impact of clubbing the 1,1′-biphenyl rings at the 5-position of a halogenated pyrazole core, revealing critical insights into the scaffold’s therapeutic index. Through meticulous pharmacophore tuning, they observed that substituting a trifluoromethyl group with a difluoromethyl moiety at the pyrazole 3-position (Compound **14**) drastically decoupled antimicrobial efficacy from mammalian toxicity. While the trifluoromethyl analog **50** (Figure 29) exhibited severe cytotoxicity (only 6.49% cell viability), the difluoromethyl derivative **14** (Figure 29) displayed exceptional safety (94.00% cell viability in 3t3L1 fibroblasts) alongside a robust, dual-action antimicrobial profile. Compound **14** exhibited potent antibacterial activity against *Bacillus subtilis* (MIC = 31.25 µg/mL) and, as seen, concurrent antifungal efficacy against *Aspergillus niger* (MIC = 31.25 µg/mL) and *Candida albicans* (MIC = 62.5 µg/mL). Mechanistically, docking and spectroscopic studies confirmed that the biphenyl-pyrazole scaffold of **14** deeply anchors into the active site of sterol 14-alpha demethylase (CYP51B, ΔG = −8.3 kcal/mol), effectively halting ergosterol biosynthesis. This highlights the biphenyl moiety’s capacity to serve as a broad-spectrum, multi-kingdom anti-infective vector when its lipophilicity is appropriately balanced to prevent off-target mammalian membrane disruption [27].

**Figure 30:**
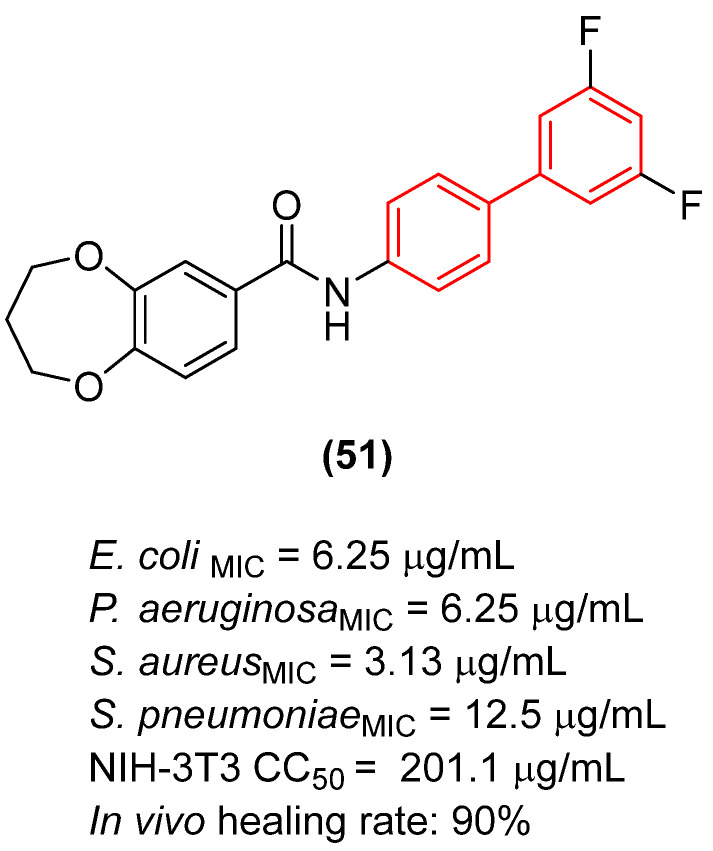
Pharmacological profiling of compound **51**.

Beyond membrane disruption, the strict biphenyl moiety has proven indispensable for specific enzyme inhibition by precisely occupying deep hydrophobic pockets. Jia et al. [59] explored the inhibition of *E. coli* FabH (β-ketoacyl-ACP synthase), a critical enzyme in the Type II fatty acid synthesis (FAS II) pathway. They demonstrated that modifying the biphenyl core with fluorine and trifluoromethyl substituents significantly modulates both binding affinity and metabolic stability. The introduction of a bis-fluorine substitution at the ortho and meta positions (Compound **51**, Figure 30) locked the dihedral conformation, yielding robust broad-spectrum activity against *E. coli* (MIC = 6.25 µg/mL) and *S. aureus* (MIC = 3.13 µg/mL) and enhancing van der Waals interactions within the FabH active site (IC_50_ = 4.1 µM). Notably, compound **51** exhibited robust in vivo antibacterial activity, promoting a 90% wound closure rate by Day 10 (versus 70% in the blank group). Subsequent histological evaluation confirmed that the experimental group effectively suppressed inflammatory cellular infiltration, in sharp contrast to the sustained inflammation observed in the untreated control [59].

**Figure 31:**
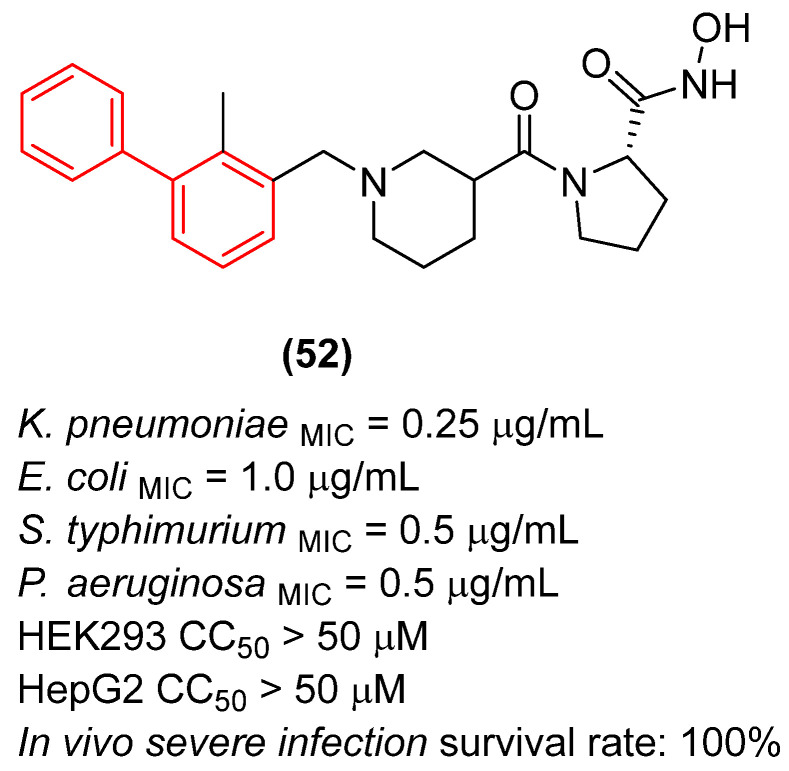
Pharmacological profiling of compound **52**.

Finally, the ability of the biphenyl structure to concurrently act as an immune checkpoint inhibitor was elegantly exploited by Zhang et al. [60] in the design of LpxC/PD-L1 dual-target inhibitors. Through a rational structural splicing approach, the biphenyl scaffold was utilized to dock precisely into the hydrophobic subpockets of the PD-L1 interface, mimicking established biphenyl-based immune inhibitors, while a linked hydroxamic acid moiety coordinated the catalytic zinc ion in the LpxC active site. The resulting lead compound (**52**, Figure 31) not only displayed excellent antibacterial activity against *Klebsiella pneumoniae* and *P. aeruginosa* (MIC = 0.25–0.5 µg/mL) and no cytotoxicity to HEK293 (Human Embryonic Kidney) and HepG2 (Hepatocellular Carcinoma) cells but, when formulated into an environment-sensing nanocarrier (NC-12d), effectively restored host antibacterial immunity in vivo, eliciting a 100% survival rate in severe murine infection models [60].

#### 2.3.1. Cross-Sectional SAR: Topological Control, Membrane Disruption, and Cell Division

A critical evaluation of the aforementioned studies reveals that the structural recalcitrance of bacterial pathogens has driven the optimization of the biphenyl scaffold far beyond its historical characterization as a simple, passive lipophilic anchor. Across diverse chemotypes, the [1,1′-biphenyl] unit functions as a conformationally tunable vector, capable of dictating the precise three-dimensional topology required for target engagement.

For Gram-positive pathogens, the scaffold’s capacity to optimize electronic distribution and structural rigidity is paramount. Wang et al. [47] established that polyhydroxylation on the B-ring, coupled with strong electron-withdrawing groups (e.g., -CF3) on the A-ring of biphenyl phytoalexins, dictates antibacterial efficacy by creating an optimal push-pull electronic configuration. Similarly, Deng et al. [7] proved that scaffold hopping from a highly flexible benzene ring to a sterically restricted biphenyl moiety in benzamide derivatives yielded a remarkable 32-fold increase in potency against *B. subtilis*. This rigidification actively targets the cell division protein FtsZ, functioning as a highly selective bactericidal agent (MBC/MIC ≤ 4) with zero observed cytotoxicity in Vero cells [7].

The requirement for structural rigidity to overcome Gram-positive resistance is further reinforced by Li et al. [58] and Demchenko et al. [24]. By utilizing a 3,3′-biphenyl linkage, quaternary ammonium peptidomimetics act as rigid spacers that prevent hydrophobic collapse, ensuring selective disruption of bacterial PG/CL membranes without compromising the integrity of mammalian erythrocytes [58]. Likewise, bulky biphenyl substituents provide the essential steric contacts required for anti-MRSA activity in imidazo[1,2-a]azepines [24].

Addressing the formidable barrier of Gram-negative pathogens requires precise topological control over facial amphiphilicity. As systematically demonstrated by Tague et al. [26], positional isomerism on the biphenyl core radically alters the antibacterial spectrum. Shifting substituents from a baseline 2,2′-arrangement to a 3,2′-configuration topologically optimizes the amphiphilic balance required to penetrate the outer membranes of *P. aeruginosa* and *E. coli*, yielding an exponential increase in efficacy [26].

#### 2.3.2. Targeted Enzyme Inhibition, Dual-Targeting, and Advanced In Vivo Antibacterial Efficacy

Beyond generalized membrane disruption, the biphenyl architecture is highly effective at occupying deep hydrophobic enzymatic pockets, a strategy successfully employed against intracellular and resilient pathogens. In the context of tuberculosis, Cheng et al. [54] utilized the biphenyl structure to target the virulence factor MptpB. The introduction of electron-withdrawing halogens on the biphenyl framework drastically increased target engagement inside infected macrophages, demonstrating the scaffold’s ability to cross complex host–pathogen barriers [54].

The active manipulation of the biphenyl dihedral angle—often termed conformational locking—represents a major structural trend. Jia et al. [59] exploited this by introducing ortho and meta bis-fluorination to the biphenyl core, restricting rotational freedom to ensure an exact fit within the active site of *E. coli* FabH (IC_50_ = 4.1 µM). This halogenation strategy concurrently shielded the molecule from rapid metabolic degradation [59]. Similarly, tethering the biphenyl ring to pyrazole cores significantly enhanced lipophilic target penetration against specific Gram-negative bacilli like *P. mirabilis* [27].

The apex of current biphenyl SAR optimization is the conceptualization of dual-target therapeutics that bridge direct pathogen inhibition with host immune modulation. Severe Gram-negative infections invariably trigger massive lipopolysaccharide (LPS) release, upregulating the PD-L1 immune checkpoint and inducing host T-cell exhaustion. Zhang et al. [60] successfully engineered dual LpxC/PD-L1 inhibitors, utilizing the strict biphenyl core to mimic PD-L1 dimerizers while a linked hydroxamic acid targets LPS biosynthesis. The translation of the lead compound (**52**) into an environment-sensing nanocarrier (NC-12d) confirms the in vivo viability of the biphenyl scaffold. By simultaneously paralyzing bacterial replication and restoring host immunity, this system elicited a 100% survival rate with no adverse systemic toxicity in murine models, robustly answering concerns regarding the in vivo safety and translational potential of biphenyl-based anti-infectives [60].

Table 9 summarizes these overarching cross-sectional structural trends, mechanisms of action, and translational profiles for biphenyl-based antibacterial agents.

### 2.4. Antiparasitic Activity

Neglected tropical diseases (NTDs) are caused by a variety of pathogens associated with social and economic conditions, affecting poorer populations predominantly in tropical and subtropical regions and causing devastating health consequences [61,62].

As estimated by the World Health Organization, more than one billion individuals are affected by these diseases, which illustrates their significant impact on global public health. The process of discovery and development of drugs to treat these diseases is insufficient, primarily due to inadequate investment by governments of developed countries and large pharmaceutical industries [61,62,63].

Chagas disease represents one of the most significant neglected tropical diseases (NTDs). The disease is caused by the protozoan *Trypanosoma cruzi* and is vectored by triatomine insects. It is estimated that approximately 6 to 7 million people worldwide are infected with *T. cruzi*, predominantly in Latin America, where it is considered an endemic disease [64,65].

The current therapeutic options for Chagas disease are limited to two drugs: benznidazole (BNZ) and nifurtimox. However, in addition to their low efficacy in patients with chronic disease, safety and tolerability issues represent a significant challenge for these chemotherapeutics. Consequently, the creation of novel, safer, and more efficacious therapeutic treatments represents a significant challenge for the control of Chagas disease.

**Figure 32:**
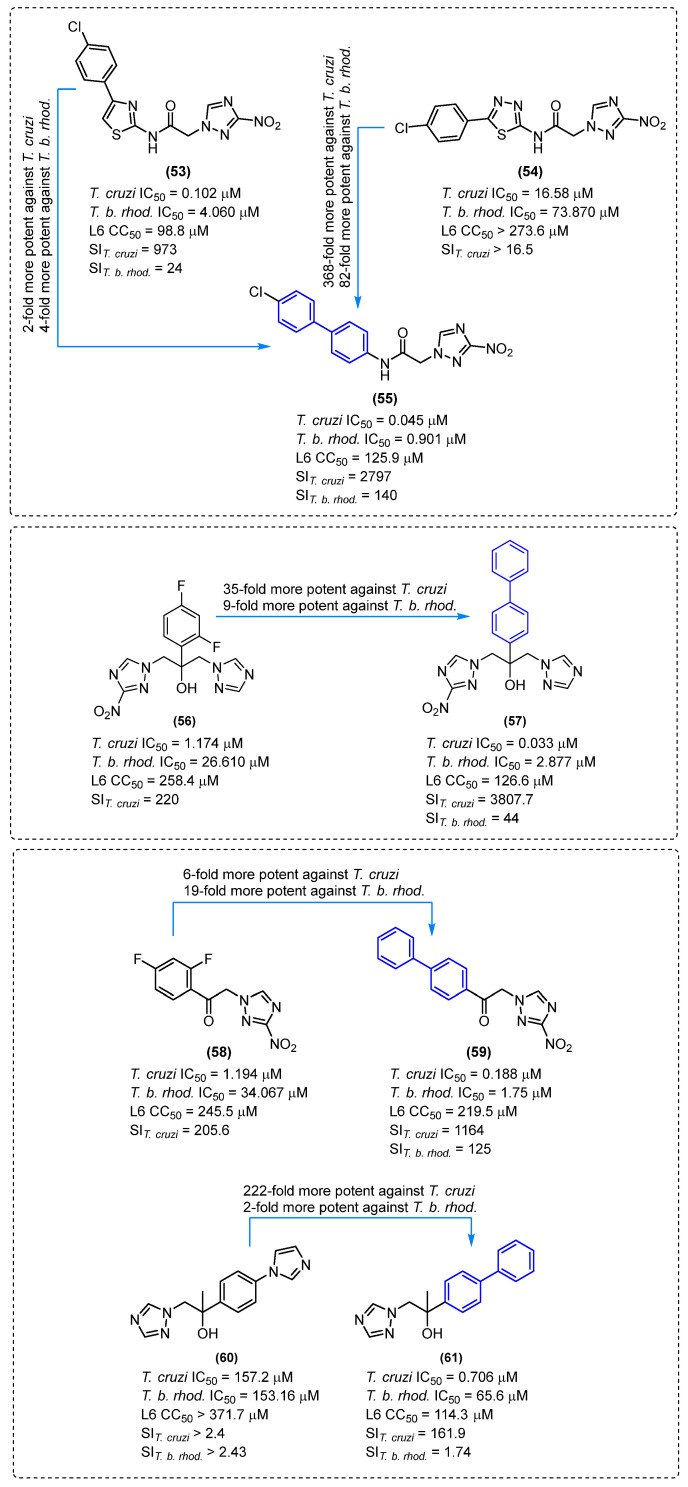
Novel biphenyl derivatives as anti-trypanosomatid agents.

In their search for new substances for the treatment of Chagas disease, Papadopolou and colleagues [66] developed a series of compounds that act via multiple mechanisms. These include acting as substrates of the *T. cruzi* nitroreductase enzyme (NTR) and as inhibitors of the CYP51 enzyme of this parasite. The antiparasitic activity was evaluated against the amastigote forms of *T. cruzi* and the blood forms of *Trypanosoma brucei rhodesiense* (*T. b. rhod.*), another trypanosomatid that is the etiological agent of sleeping sickness, another important NTD. Moreover, the cytotoxicity of these compounds was evaluated in rat skeletal L6 myoblasts. Among the synthesized derivatives, particular mention may be made of the biphenyl derivatives **55**, **57**, **59** and **61**, which were classified as active against *T. cruzi*, with IC_50_ values of 0.045 µM, 0.188 µM, 0.033 µM and 0.706 µM, respectively (Figure 32). Regarding *T. b. rhod*., these derivatives were deemed to exhibit moderate activity, as evidenced by their IC_50_ values falling within the range of 0.5 to 6.0 µM (Figure 32). It was observed that the incorporation of biphenyl resulted in an enhancement of the antiparasitic activity of the derivatives against *T. cruzi* and *T. b. rhodesiense*. Derivative **55** exhibited approximately two-fold and 368-fold greater potency against *T. cruzi* than derivatives **53** and **54**, respectively. Additionally, it exhibited approximately 4-fold and 82-fold greater potency against *T. b. rhod.* in comparison to derivatives **53** and **54**, respectively (Figure 32). The replacement of the phenylimidazole nucleus of **60** by biphenyl resulted in a notable enhancement in activity against *T. cruzi* and *T. b. rhod*., with derivative **61** exhibiting approximately 222- and 2-fold increase in potency against these parasites, respectively (Figure 32) [66].

The replacement of the difluorobenzene ring with a biphenyl moiety led to a notable enhancement in antiparasitic activity. Derivative **57** exhibited approximately sixfold and 19-fold greater potency than **56** against *T. cruzi* and *T. b. rhod*., respectively. Similarly, derivative **57** demonstrated approximately 35-fold and 9-fold greater potency than 56 against *T. cruzi* and *T. b. rhod*., respectively (Figure 31) [66].

**Figure 33:**
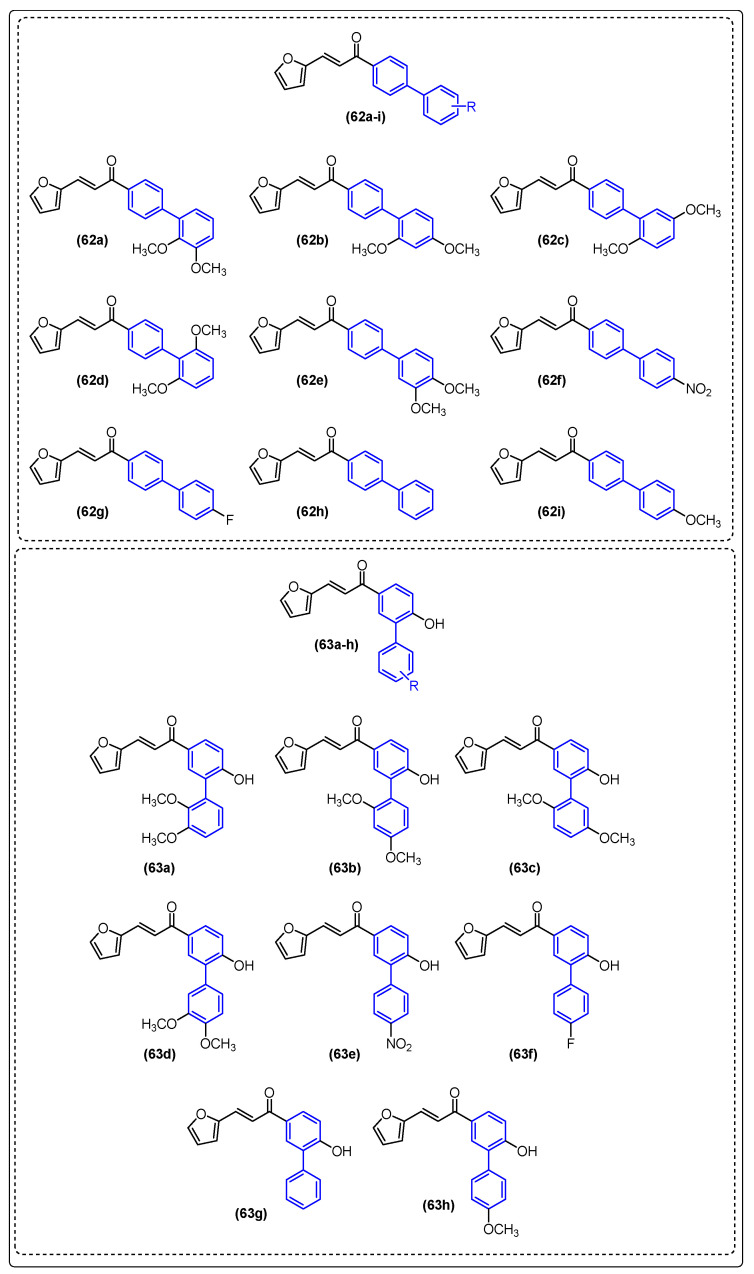
New furanochalcone-biphenyl hybrids.

Garcia and colleagues [67] synthesized a series of furanochalcone-biphenyl hybrids (**62a–i** and **63a–i**, Figure 33) and evaluated their antitrypanosomal activities in vitro, as well as their cytotoxicity, using a standardized in vitro toxicity assay (Figure 32).

Of the 17 furanochalcone-biphenyl hybrids synthesized, compounds **62a–d**, **63a–f**, and **63h** demonstrated activity against intracellular amastigotes of *T. cruzi*, exhibiting EC_50_ values between 10.52 and 30.41 µM (Table 10). Compound **63e** exhibited the highest level of activity within the series, with an EC_50_ value of 10.52 ± 0.23 µM. This level of activity was superior to that of BNZ, a drug utilized as positive control (EC_50_ = 40.3 ± 6.92 µM) [67].

Leishmaniasis represents a significant public health concern, with a disproportionate impact on developing countries. As estimated by the World Health Organization, approximately 700,000 to 1 million cases occur annually [68]. It is an NTD caused by several pathogenic species of the genus *Leishmania*, which can present in a range of forms, from benign skin lesions to visceral infections that can ultimately result in the death of the patient.

The necessity for new therapeutic strategies to combat and control leishmaniasis is evident, given the limitations of the current treatment options. These include the high toxicity of existing treatments, their high cost, the emergence of drug-resistant strains, and the challenges posed by co-infections such as HIV/*Leishmania* spp. These factors have collectively compromised the control and treatment of this disease [69,70].

**Figure 34:**
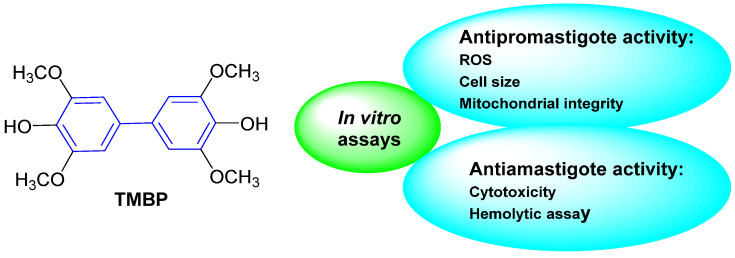
Pharmacological properties of the compound TMBP.

Schirmann and colleagues [71] conducted a study to investigate the biological activity of the compound 3,3′,5,5′-tetramethoxy-biphenyl-4,4′-diol (TMBP) (Figure 34) against the amastigote and promastigote forms of *L. amazonensis*. Previously, TMBP has been demonstrated to possess anticancer activity through its ability to increase the production of reactive oxygen species, which ultimately leads to cellular apoptosis [71].

Following a 24 and 48 h incubation period, the viability of the parasites was significantly reduced by TMBP, indicating potential antileishmanial activity. The antileishmanial effect of the TMBP compound was confirmed through a flow cytometry analysis conducted over a 24 h period, indicating a reduction in the cell size of promastigotes at concentrations ranging from 1.95 to 15.6 µM. This finding suggests that the observed effect may be apoptosis [71].

Maintaining mitochondrial integrity is a crucial aspect of parasite survival. Subsequently, the potential mechanisms underlying parasite death were investigated following confirmation of the antileishmanial effect of the compound. This involved an evaluation of the production of reactive oxygen species (ROS) and mitochondrial integrity. In this analysis, TMBP concentrations of 0.86 µM (IC_50_) and 1.72 µM (twice the IC_50_ value) were employed. The total fluorescence intensity of TMRE (the probe utilized to assess the potential alteration of mitochondrial integrity induced by TMBP) was observed to diminish in comparison to the control group, indicating that TMBP may exert an impact on the mitochondrial integrity of the parasite [71].

The production of reactive oxygen species (ROS) was also evaluated at the same concentrations as in the previous test. It was observed that the treated parasites demonstrated an increase in ROS levels when compared to the control group [71].

The cytotoxic action of TMBP was evaluated in two mammalian cells: murine macrophages and sheep erythrocytes. The concentrations ranged from 0.03 µM to 15.60 µM, and the CC_50_ value was determined to be 53.93 µM. Additionally, no hemolysis was observed in erythrocytes at the concentrations tested [71].

Malaria is an infectious disease caused by five species of parasites belonging to the genus *Plasmodium* [72]. Of these, *P. falciparum* and *P. vivax* represent the species that pose the greatest threat. In 2022, approximately 249 million cases and approximately 608 thousand deaths from malaria were reported worldwide, according to data from the World Health Organization (WHO) [72].

A significant challenge in the treatment and control of this disease is the emergence of drug resistance. Hybrid molecules occupy a significant position in the field of medicinal chemistry. Molecular hybridization is an effective tool in the process of drug discovery and development. This approach involves the joining of two pharmacophoric or bioactive subunits to create a new chemical entity, with the aim of enhancing efficacy, improving affinity, and optimizing pharmacokinetic and pharmacodynamic properties. This strategy has the potential to reduce toxicity and enhance the therapeutic index, while also combating the global problem of drug resistance [73,74].

**Figure 35:**
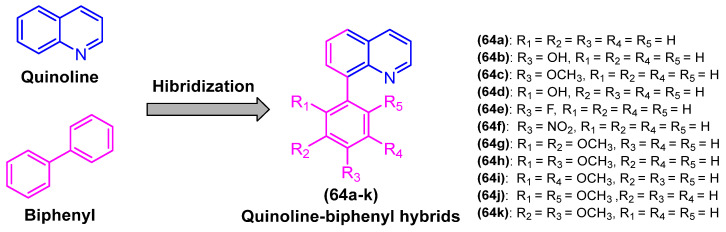
Design of new quinoline-biphenyl hybrids.

In a study conducted by Coa and colleagues [75], eleven quinoline-biphenyl hybrids (Figure 35) were synthesized and evaluated against *P. falciparum* and amastigote forms of *Leishmania panamensis* and *T. cruzi* as part of the search for new substances for the treatment of cutaneous leishmaniasis, Chagas disease, and malaria. The quinoline nucleus was selected for its association with notable antiparasitic activity, particularly against *Plasmodium* species, given the prevalence of quinoline derivatives in antimalarial medications.

**Figure 36:**
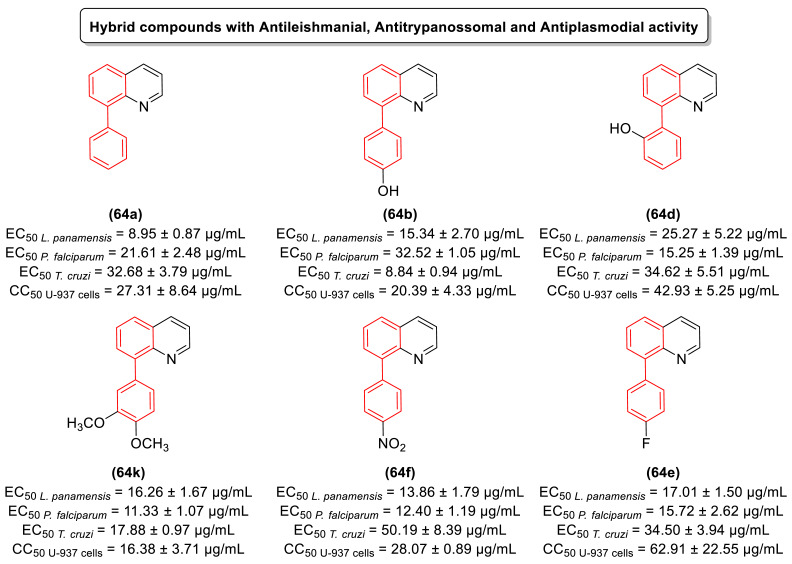
Pharmacological properties of new quinoline-biphenyl hybrids.

All compounds were subjected to in vitro evaluation for antileishmanial, antitrypanosomal, and antiplasmodial activity, in addition to cytotoxicity in human U-937 macrophages. The results demonstrated that compounds **64a**, **64b**, **64e**, **64f**, and **64k** exhibited efficacy against *L. panamensis* amastigotes, with EC_50_ values lower than 20 µg/mL. Of these, the hybrid **64a** exhibited the most notable activity, with an EC_50_ value of 8.95 ± 0.87 µg/mL, which was comparable to that of the reference drug meglumine antimoniate (EC_50_ 9.4 ± 2.1 µg/mL). Hybrids **64b** and **64k** demonstrated efficacy against intracellular amastigotes of *T. cruzi*, with compound **64b** exhibiting an EC_50_ value of 8.84 ± 0.94 μg/mL, comparable to that of benznidazole (EC_50_ = 10.5 ± 1.8 μg/mL). The compounds with the most favorable in vitro activity against *P. falciparum* were **64d**, **64f**, and **64k**, with hybrid **64k** exhibiting the highest level of activity with an EC_50_ value of 11.33 ± 1.07 μg/mL (Figure 36). In the cytotoxicity evaluation in U-937 human macrophages, only compounds **64c**, **64g**, and **64h** exhibited moderate cytotoxicity, with LC_50_ values exceeding 100 μg/mL [75].

**Figure 37:**
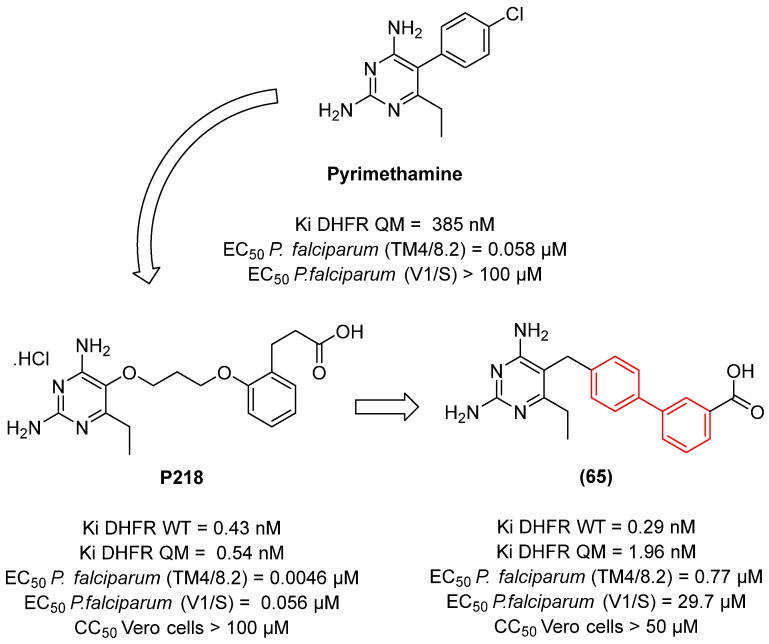
Pharmacological profiling of compound **65**.

A recent study by Hoarau et al. [76] provides a compelling example of using a fragment-based drug design (FBDD) strategy to overcome pyrimethamine resistance in Plasmodium falciparum dihydrofolate reductase (PfDHFR). In an effort to optimize the pharmacokinetic profile of the clinical candidate P218 (an improved analogue of the antimalarial pyrimethamine, Figure 37), researchers replaced a metabolically labile, flexible phenyl propanoate moiety with a rigid bi-aromatic carboxylate scaffold, utilizing the biphenyl core to enhance lipophilicity and structural rigidity. This scaffold was selected because fragment screening revealed exceptionally slow dissociation rates (K_off_) and an optimal topological fit within the para-aminobenzoic acid (PABA) binding pocket of the quadruple mutant (QM) PfDHFR [76].

The structural rigidity of the strict biphenyl core served to maintain a precise geometric vector between the 2,4-diaminopyrimidine anchoring “head” and the carboxylate “tail”. Through an extensive structure-activity relationship (SAR) investigation evaluating various connectivity patterns (3,4′-, 4,3′-, 3,3′-, and 4,4′-), the 3,3′-linked derivative was identified. High-resolution co-crystallographic studies (e.g., PDB 8JFD) demonstrated that the biphenyl architecture optimally occupies the hydrophobic cleft while restricting the torsion angle to correctly orient the terminal carboxylate, forming critical hydrogen bonds with active site residues Arg59 and Arg122 [76].

From a critical medicinal chemistry perspective, however, this series highlights the dual-edged nature of incorporating unadulterated biphenyl systems. While the strict biphenyl core drove profound target engagement and conferred complete metabolic stability against phase 1 liver microsomes, the resulting excessive lipophilicity and molecular rigidity caused a dramatic reduction in kinetic solubility at physiological pH. Consequently, despite exceptional enzymatic inhibition, the cellular antimalarial efficacy was disappointing. In addition, the most active derivative in whole-cell assays (Compound **65**, Figure 37) exhibited an IC_50_ of 0.77 µM against the sensitive *P. falciparum* TM4/8.2 strain but lost significant potency against the resistant V1/S strain (IC_50_ = 29.7 µM). This translates to a massive discrepancy between the enzymatic K_i_ and the cellular IC_50_. This case study underscores a fundamental SAR principle for biphenyl-based drug discovery: while the scaffold acts as a powerful, metabolically stable pharmacodynamic anchor, its rigid and highly hydrophobic nature must be delicately balanced with polar or ionizable solubilizing appendages (especially basic motifs) to ensure adequate membrane permeability and in vivo translation [76].

**Figure 38:**
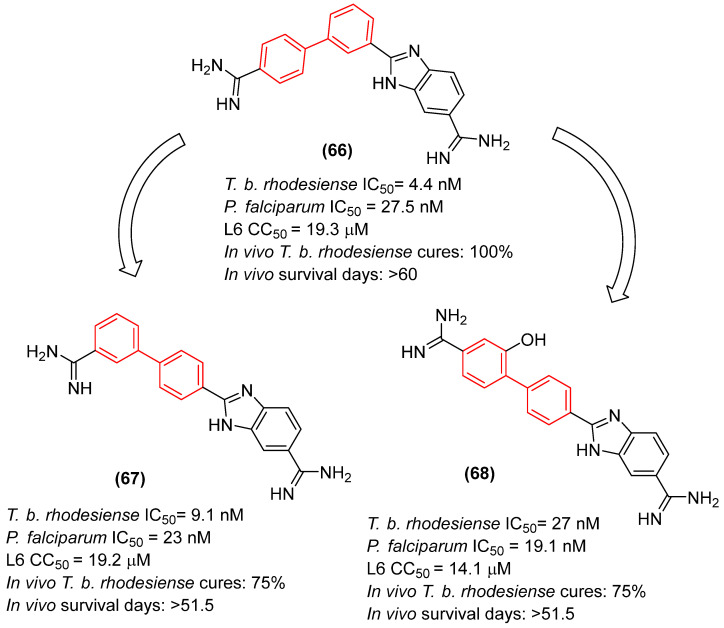
Pharmacological profiling of compound **66**–**68**.

The structural and pharmacological analysis of the work by Ismail et al. [77] reveals the biphenyl core not merely as a lipophilic anchor, but as a strictly restricted topological element essential for molecular recognition within the DNA minor groove. This study represents a significant milestone in the medicinal chemistry of antiprotozoal agents, detailing the rational design of a series of dicationic benzimidazole derivatives linked by a central biphenyl bridge, which were strategically designed utilizing compound **66** as the structural starting point (Figure 38).

The main point of the Structure-Activity Relationship (SAR) in this study lies in the geometric exploitation of the 4,4′-biphenyl axis. In contrast to *ortho* or *meta* arrangements that would inherently induce a kink in the molecule, the *para*, *para*′ (4,4′) substitution generates a near-linear, extended, and rigid architecture. This geometric linearity is strictly required for topological complementarity: the specific spatial distance and the slight natural torsional twist of the biphenyl molecule perfectly match the architecture of the minor groove of adenine-thymine (AT)-rich DNA sequences, which are prominent in the kinetoplast DNA (kDNA) of trypanosomatids and the genome of *Plasmodium* species [77].

The terminal pharmacophoric groups (amidines or benzimidazoles) operate synergistically with the biphenyl spacer. At physiological pH, these functional groups are protonated, conferring a permanent dicationic character to the molecule. This dual positive charge provides the electrostatic driving force for initial attraction to the polyanionic DNA sugar-phosphate backbone. The rigid spacing guaranteed by the biphenyl core ensures that, once inserted deep into the minor groove, the terminal heteroatoms are perfectly vectored to establish maximized van der Waals interactions and dense hydrogen-bonding networks with the nitrogenous bases at the floor of the groove, thereby highly stabilizing the ligand-DNA complex and stalling parasitic replication [77].

Translating this structural refinement to in vitro and in vivo models attests to the remarkable robustness of the design. The synthesized biphenyl diamidines displayed potent in vitro activity against two major protozoan parasites, exhibiting IC_50_ values ranging from 3 to 37 nM against *Trypanosoma brucei rhodesiense* and an even more exceptional potency against *Plasmodium falciparum*, with IC_50_ values ranging from 0.5 to 23 nM. Importantly, this profound antiprotozoal activity was accompanied by low in vitro cytotoxicity against mammalian host cells (L6 rat myoblasts), thereby ensuring a highly favorable selectivity index and underscoring the target-specific nature of the DNA minor groove binding [77].

In in vivo efficacy evaluations using the STIB900 murine model for acute African trypanosomiasis via *Trypanosoma brucei rhodesiense* infection, the highly optimized derivatives, specifically compounds **67** and **68** (Figure 38), emerged as the most promising lead molecules. These compounds achieved a remarkable 3/4 cure rate at an intraperitoneal dosage of 20 mg/kg, maintaining a robust safety margin [77].

However, from a rigorous clinical development perspective, it is mandatory to acknowledge the pharmacokinetic hurdles inherent to this chemical class. The permanent dicationic character, while indispensable for pharmacodynamic target inhibition and active cellular sequestration by the parasite’s transport systems, imposes severe limitations on passive transcellular permeability in the mammalian gastrointestinal tract. Consequently, the high polar surface area at the extremities, juxtaposed with the highly lipophilic central biphenyl core, generally results in poor oral bioavailability. This critical bottleneck restricts administration primarily to parenteral routes, necessitating sophisticated formulation approaches or the rational design of prodrugs (e.g., amidoximes) to effectively circumvent this pharmacokinetic barrier and unlock their full translational potential.

#### Cross-Sectional SAR and In Vivo Antiparasitic Activity

The exploration of the biphenyl core in the design of antiparasitic agents has proven to be a formidable, albeit complex, strategy to circumvent the inherent challenges of treating neglected tropical diseases and malaria. A critical analysis of cross-sectional Structure-Activity Relationship (SAR) trends reveals that the biphenyl scaffold predominantly acts through two distinct mechanistic roles: as a rigid conformational vector and as a highly lipophilic anchor. In the therapeutic landscape of Chagas disease and leishmaniasis, the replacement of simple or difluorinated benzene rings with the strict biphenyl core has drastically optimized spatial filling within deep hydrophobic enzymatic active sites. This is notably evidenced by the bifunctional CYP51 and nitroreductase inhibitors of *Trypanosoma cruzi* developed by Papadopoulou et al. [66], where precise steric adjustments and optimized dihedral angles dictated the exponential increase in antiprotozoal potency.

Simultaneously, molecular hybridization strategies have demonstrated that inserting the biphenyl unit as a lipophilic bridge not only facilitates cellular penetration into host macrophages but also confers broad-spectrum target engagement. The furanochalcone-biphenyl hybrids proposed by Garcia et al. [67] and the quinoline-biphenyl chimeras developed by Coa et al. [75] exemplify how this robust scaffold can rescue failing pharmacophores that, in isolation, would be unable to reach the intracellular amastigote forms of *T. cruzi* and *Leishmania panamensis*. Beyond its utility as a mere spacer, the intrinsic bioactivity of the unadulterated biphenyl core was unequivocally validated by Schirmann et al. [71]; the polyoxygenated derivative TMBP was shown to act directly in inducing apoptosis in *Leishmania amazonensis* via mitochondrial collapse and a massive burst of reactive oxygen species (ROS), all while maintaining a highly selective safety profile devoid of hemolytic toxicity against mammalian cells.

However, the translational application of this structural privilege requires extreme caution in managing physicochemical properties. The recent work by Hoarau et al. [76] on the inhibition of *Plasmodium falciparum* dihydrofolate reductase (PfDHFR) illustrates a classic “brick dust” paradigm in medicinal chemistry. By utilizing the biphenyl moiety to rigidly anchor a 2,4-diaminopyrimidine ring deep into the para-aminobenzoic acid (PABA) pocket, the authors achieved sub-nanomolar inhibition against both wild-type and quadruple mutant (QM) strains of the enzyme, alongside excellent stability against cytochrome P450-mediated phase 1 metabolism. Nevertheless, the excessive molecular rigidity and high lipophilicity induced by the strict biphenyl system caused a sharp decline in kinetic solubility at physiological pH. Consequently, a severe translational gap was observed: molecules that were brilliantly active in enzymatic assays (K_i_) drastically lost efficacy in whole-cell assays (IC_50_) against the parasite.

Conversely, when the conformational vector of the biphenyl core is exploited in targets demanding strict linear topologies—such as the 4,4′-substituted dicationic diamidines described by Ismail et al. [77] for binding to the minor groove of kinetoplast AT-rich DNA—important in vivo efficacy is observed. This precise geometric alignment allows these derivatives to achieve sterile cures in murine models of *Trypanosoma brucei rhodesiense* infection at very low doses (0.20 mg/kg), significantly outperforming standard clinical drugs with a remarkably wide therapeutic window.

Therefore, the clinical utility of the biphenyl core as an antiparasitic agent relies heavily on an iterative fine-tuning process. It is imperative to maximize hydrophobic and van der Waals contacts for pharmacodynamic gain, without neglecting the introduction of polar, ionizable appendages or ring symmetry modifications to prevent aqueous aggregation and non-specific cytotoxicity. Table 11 critically summarizes these cross-sectional structural trends and their associated pharmacokinetic successes and bottlenecks across the main classes of recent antiparasitic biphenyl derivatives.

### 2.5. Clinical Translation of the Biphenyl Scaffold: FDA-Approved Anti-Infective Drugs

The definitive validation of the biphenyl subunit as a privileged scaffold in medicinal chemistry relies on its successful translation from preclinical models to FDA-approved therapeutics targeting viral, bacterial, and fungal pathogens. In these advanced agents, the biphenyl core transcends the role of a mere hydrophobic filler; it assumes vital pharmacodynamic functions, acting as a precise topological spacer, a thermodynamic membrane anchor, and a strict conformational restrictor.

**Figure 39:**
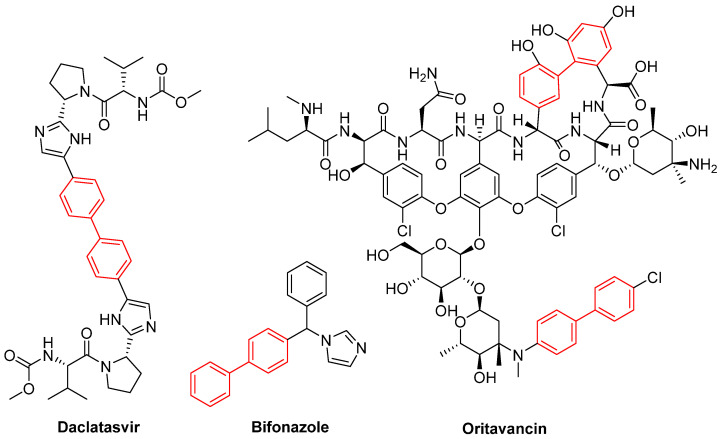
FDA-Approved Biphenyl Anti-Infective Drugs.

In the landscape of viral infections, daclatasvir (BMS-790052) (Figure 39) represents a state-of-the-art rational design anchored by a biphenyl scaffold. Approved as a first-in-class, pangenotypic direct-acting antiviral (DAA) for the Hepatitis C virus (HCV), the molecule’s architecture masterfully exploits the intrinsic C_2_-symmetry of the [1,1′-biphenyl]-4,4′-diyl core. Within this context, the scaffold acts as a perfectly rigid and geometrically calibrated topological vector. The rotational restriction of the biphenyl axis, combined with its linear length, projects the capping pharmacophores (L-prolyl-L-valine derivatives) at the exact steric distance required to simultaneously occupy the symmetric clefts of the dimeric viral NS5A protein interface. This bivalent blockade, which is strictly dependent on the rigidity and precise length of the biphenyl core, halts the assembly of the viral replication complex, conferring the drug an extraordinary inhibitory potency in the picomolar range—a thermodynamic feat impossible to achieve with flexible aliphatic or monoregional spacers [78,79].

The profound impact of the biphenyl moiety in overcoming complex bacterial resistance mechanisms is exemplified by oritavancin (Figure 39), a second-generation lipoglycopeptide approved for acute multiresistant Gram-positive infections, including Methicillin-resistant *Staphylococcus aureus* (MRSA) and Vancomycin-resistant Enterococci (VRE). The critical semi-synthetic modification relative to natural vancomycin is the alkylation of the central disaccharide amine with a robust 4′-chloro-biphenyl-4-ylmethyl side chain. Extensive SAR analyses reveal that this halogenated domain operates as a bifunctional vector. Primarily, the highly lipophilic biphenyl system acts as a massive anchor that deeply intercalates into the bacterial cell membrane bilayer, severely disrupting membrane integrity and causing rapid depolarization—a secondary lethal mechanism of action. Even more critically, the thermodynamic anchoring generated by the biphenyl moiety drastically increases the local residence time and promotes drug dimerization. This strong entropic compensation allows oritavancin to maintain its binding affinity even when the bacterial peptidoglycan precursor mutates from D-Ala-D-Ala to D-Ala-D-Lac (as seen in VanA/VanB phenotypes), brilliantly overcoming the critical loss of a hydrogen bond that otherwise renders classical vancomycin obsolete [80,81].

In the field of medical mycology, broad-spectrum dermatological agents like bifonazole (Figure 39) demonstrate the utility of the biphenyl system in promoting steric occlusion within enzymatic active sites. Unlike systemic small-molecule triazoles, bifonazole features a bulky 1-([1,1′-biphenyl]-4-yl(phenyl)methyl) group attached to an imidazole ring. Mechanistic docking and crystallographic studies suggest that the extended biphenyl axis projects deeply into the narrow hydrophobic access channel of the fungal lanosterol 14-α-demethylase (CYP51). This massive steric bulk restricts the rotational degrees of freedom of the molecule within the cavity, forcing the free nitrogen (N_3_) of the azole ring to maintain a perfectly orthogonal and uninterrupted coordination geometry with the prosthetic heme iron. Beyond optimizing catalytic inhibition, the imposed spatial volume of the biphenyl dictates an extreme lipophilicity (log P > 4.5), which translates into exceptional pharmacokinetic retention within the human stratum corneum. This localized thermodynamic distribution is fundamentally responsible for the clinical efficacy of bifonazole as a single-application topical therapy [82,83].

## 3. Conclusions

The strict biphenyl scaffold has irrefutably cemented its status as a privileged structure in modern medicinal chemistry. Moving far beyond its historical characterization as a simple, passive lipophilic filler, the true biphenyl moiety is now recognized as a highly directional, conformationally tunable architectural vector. The integration of advanced computational conformational analysis into rational drug design has revealed that the profound thermodynamic and entropic penalties associated with target binding can be heavily mitigated. By strategically locking the biphenyl dihedral angle through ortho-substitutions, researchers can generate highly selective, rigidified topographies that perfectly complement the geometry of complex biological targets. This is evidenced by the evolution of highly potent, mutation-resistant HIV-1 DAPY derivatives and sub-nanomolar SARS-CoV-2 Mpro inhibitors.

Furthermore, the unprecedented acceleration in drug discovery has witnessed the evolution of strictly biphenyl-based therapeutics from conventional single-target entities into sophisticated, multi-modal agents. The genesis of dual LpxC/PD-L1 inhibitors exemplifies the future frontier of anti-infective therapy: utilizing a single engineered biphenyl molecule to simultaneously paralyze pathogen replication while overriding the infection-induced suppression of the host’s immune system. Similarly, the strategic incorporation of the biphenyl core into molecular hybrids has continuously rescued failing pharmacophores against neglected tropical diseases.

Crucially, modern structural engineering has successfully divorced the therapeutic biphenyl from the toxicological liabilities of historical industrial polychlorinated biphenyls. With agents like Daclatasvir achieving widespread clinical application, and rationally designed compounds demonstrating pristine in vivo safety profiles and excellent oral bioavailability in advanced preclinical models, the true [1,1′-biphenyl] moiety stands validated not merely as a theoretical chemical tool, but as a clinically translatable cornerstone. As the global crises of multidrug-resistant bacteria, elusive fungal pathogens, and mutating viruses continue to escalate, future research must continue to leverage high-resolution structural biology, continuous SAR mapping, and in vivo translation models to fully realize the therapeutic potential of this remarkably adaptable scaffold in the next generation of life-saving anti-infective medicines.

## Figures and Tables

**Table 1:** In vitro antifungal activities of the most potent compounds **1a–v** (MIC, μg/mL).

*Compd.*	*R*_1_	*R*_2_	*R*_3_	*C. alb. (I)*	*C. alb. (II)*	*C. trop.*	*C. neo.*	*A. fum.*
***1i***	3,5-F	H	–CH(CH_3_)_2_	0.5	2	0.5	>16	>16
***1j***	3,5-F	H	–CH_2_CH(CH_3_)_2_	0.5	2	2	>16	>16
***1m***	2-F	H	–CH(CH_3_)_2_	0.5	0.03125	0.03125	8	2
***1n***	2-F	H	–CH_2_CH(CH_3_)_2_	0.25	0.03125	0.03125	>16	4
***1o***	3-F	H	–CH(CH_3_)_2_	0.25	0.03125	0.03125	>16	4
***1p***	3-F	H	–CH_2_CH(CH_3_)_2_	0.25	0.03125	0.03125	>16	8
***1s***	3-F	2-F	–CH(CH_3_)_2_	0.5	0.125	0.25	>16	>16
***1t***	3-F	2-F	–CH_2_CH(CH_3_)_2_	0.5	0.125	0.25	>16	>16
***1u***	2-F	2-F	CH(CH_3_)_2_	0.0625	0.03125	0.03125	8	>16
***1v***	2-F	2-F	–CH_2_CH(CH_3_)_2_	0.25	0.0625	0.0625	>16	>16
*FCZ*	-	-	-	0.5	1	1	4	>16
*ITZ*	-	-	-	0.0625	0.25	0.5	1	4

Abbreviations: *C. alb*. (I), *Candida albicans* (ATCC SC5314); *C. alb*. (II), *Candida albicans* (CPCC400523); *C. neo*., *Cryptococcus neoformans* (cgmcc 2.3161); *A. fum*., *Aspergillus fumigatus* (cgmcc 3.7795); *C. trop*., *Candida tropicalis* (cgmcc 2.3739); FCZ: Fluconazole; ITZ: Itraconazole.

**Table 2:** In vitro antifungal activities of compounds **1m**, **1o**, **1u** and **1v** (MIC, μg/mL).

*Compd*.	*R*_1_	*R*_2_	*R*_3_	*C. albicans* *strain 100*	*C. albicans* *strain 300*
***1m***	2-F	H	–CH(CH_3_)_2_	8	2
***1o***	3-F	H	–CH(CH_3_)_2_	16	4
***1u***	2-F	2-F	CH(CH_3_)_2_	16	2
***1v***	2-F	2-F	–CH_2_CH(CH_3_)_2_	8	4
*Fluconazol*	-	-	-	>64	>64

**Table 3:** Consolidated Structure-Activity Relationship (SAR) insights and toxicological profiles of recently reported biphenyl antifungal agents.

Lead Chemotype Modification	Susceptible Fungal Species	Primary Mechanism	Key Structural, SAR & Toxicological Attributes
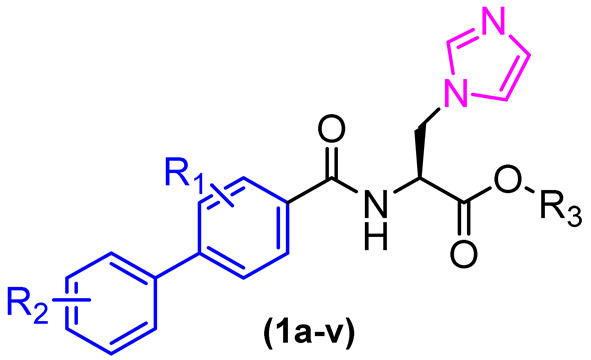 Biphenyl-Imidazole Derivatives	*Candida* spp., *Cryptococcus* spp.	CYP51 Inhibition	*Ortho*-fluorination on the biphenyl core induces an optimal dihedral twist, significantly enhancing binding affinity to the mutant CYP51 pocket and overcoming fluconazole resistance.
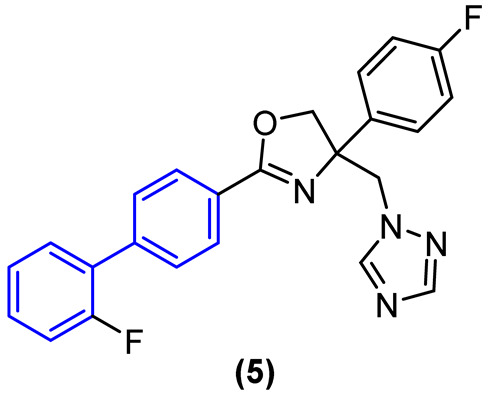 Triazole/Dihydrooxazole-Biphenyl Hybrids	*Candida* spp.	CYP51 Inhibition	Scaffold hopping (imidazole to triazole) combined with electro-reductive groups improves metabolic stability (shielding against Phase I oxidation) while maintaining sub-microgram MICs.
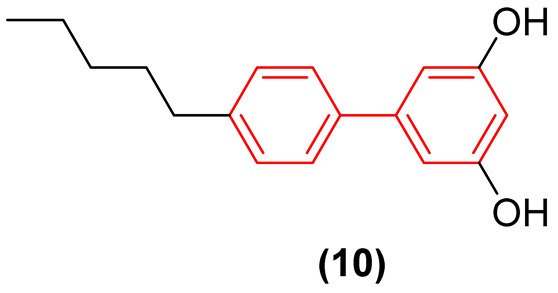 Phytoalexin-Inspired Biphenyls (Noraucuparin derivatives)	Broad-spectrum	Cell Membrane Disruption	Systematic hydroxyl/halogen substitution on the dual benzene rings increases potency up to 256-fold over the natural parent compound, acting as a highly efficient lipophilic membrane anchor with a favorable mammalian safety profile.
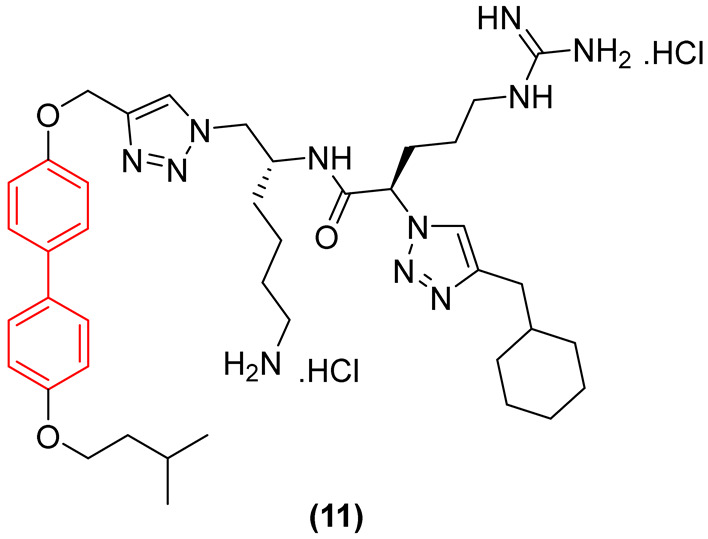 Positional Isomers of Biphenyl Peptidomimetics	*Candida albicans*	Membrane Permeabilization	The 2,2′-disubstitution pattern strictly modulates the amphiphilic topology. The 4,4′-substitution pattern is specifically optimal against *C. albicans* (MIC = 1 µg/mL), demonstrating how the biphenyl core directs the spatial vector of lipophilic/hydrophilic domains.
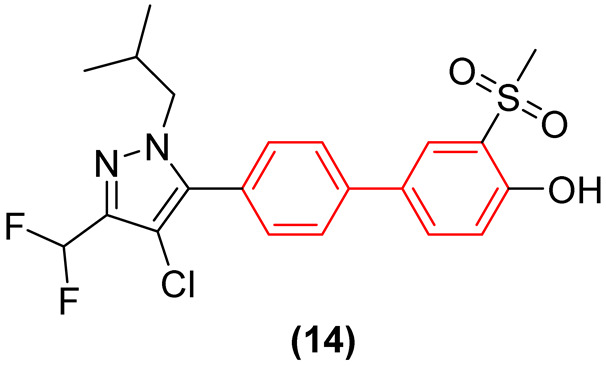 Clubbed 1,1′-Biphenyl-Pyrazole Derivatives	*Candida* spp., *Aspergillus* spp.	CYP51 Inhibition	4′-substitutions on the biphenyl ring (e.g., hydroxyl or alkoxy amino-alcohol groups) optimize the coordination of the pyrazole nitrogen with the CYP51 heme iron. Strict structural tuning yields potent antifungal activity while preserving low cytotoxicity in mammalian cells.
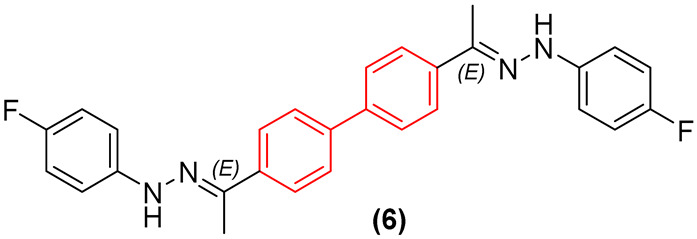 N,N′-Diaryl-bishydrazones	Broad-spectrum (Yeasts and Filamentous Fungi)	Fungistatic	Symmetrical 4,4′- vs. unsymmetrical 3,4′-biphenyl pattern dictates potency depending on alkyl substituents. Alkoxy-aryl modifications completely eliminate hERG channel toxicity and maintain excellent mammalian cell viability.
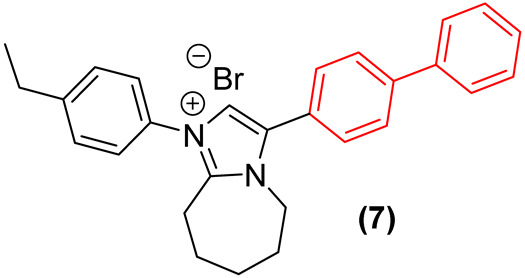 3-Biphenyl-3H-imidazo[1,2-a]azepines	*C. albicans*, *C. neoformans*	Target unconfirmed	The biphenyl core must be fused to a 7-membered azepine ring; replacing it with 6-membered rings abolishes activity. Potent antifungal effects are noted, but inherent mammalian cytotoxicity (low CC_50_ values) necessitates further structural optimization.

**Table 4:** Summary of cross-sectional SAR trends and translational impact of strict biphenyl antifungal agents.

Structural Modification/Chemotype	Primary Pathogen Target	Key SAR & Conformational Impact	In Vivo Efficacy & Pharmacokinetics
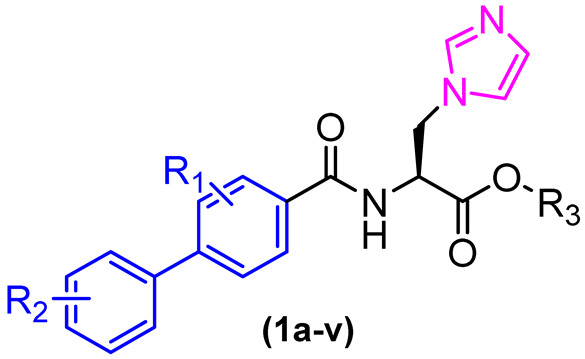 Ortho-Fluorinated Imidazole-Biphenyls	*Candida albicans*/CYP51	*Ortho*-fluorination restricts the dihedral angle for optimal CYP51 fit; active against fluconazole-resistant strains.	Favorable cytotoxicity profile (IC_50_ < 50 µM in mammalian cells); low CYP inhibition.
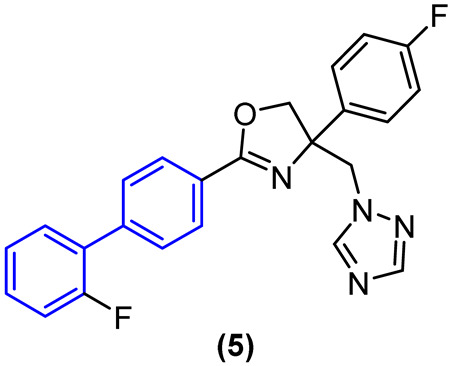 Triazole-Biphenyls with Electro-reductive Groups	*C. albicans*, *C. tropicalis*	Scaffold hopping (imidazole to triazole) combined with electronic modification of the biphenyl reduces susceptibility to Phase I oxidation.	Exceptional metabolic stability; human liver microsomal half-life increased to >145 min.
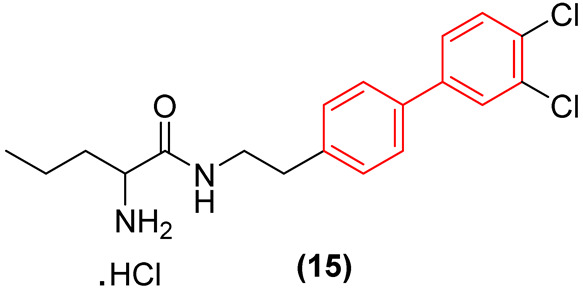 Halogenated Biphenyl Pentanamides	*C. albicans*, *C. neoformans*	Terminal *meta*/*para* dichlorination enhances lipid bilayer penetration and provides metabolic shielding.	Demonstrated robust in vivo fungicidal efficacy and systemic clearance in murine subcutaneous infection models.

**Table 5:** Inhibitory activity of selected compounds against clinically relevant HIV-1 mutant strains and WT HIV-1 RT.

	*EC*_50_ (mmol/L)							*IC*_50_ (µmol/L)
Compd.	L100I	K103N	Y181C	Y188L	E138K	F227L + V106A	K103N + Y181C	WT RT
**19a**	0.021 ± 0.015	0.016 ± 0.0071	0.047 ± 0.019	0.083 ± 0.015	0.038 ± 0.014	34.30 ± 4.12	0.074 ± 0.03	0.10 ± 0.017
**19b**	0.016 ± 0.0043	0.018 ± 0.0048	0.083 ± 0.015	0.45 ± 0.059	0.031 ± 0.016	30.84 ± 5.86	0.289 ± 0.15	0.045 ± 0.0058
**19c**	0.072 ± 0.0082	0.040 ± 0.016	0.14 ± 0.060	0.99 ± 0.28	0.052 ± 0.0096	>35	0.91 ± 0.25	0.077 ± 0.012
**17**	5.30 ± 1.49	7.78 ± 1.97	5.54 ± 1.45	104.24 ± 81.14	4.51 ± 0.37	>35	114.62 ± 58.88	3.21 ± 0.80
**DOR**	0.0066 ± 0.0017	0.042 ± 0.0013	0.025 ± 0.0023	0.50 ± 0.15	0.0075 ± 0.0026	17.35 ± 6.99	0.142 ± 0.0569	0.044 ± 0.005

EC_50_: The effective concentration required to protect MT-4 cells against viral cytopathicity by 50%. IC_50_: Inhibitory concentration of test compounds required to inhibit WT HIV-1 RT activity by 50%. DOR: Doravirine.

**Table 6:** Antiviral evaluation of compounds **23a–d**, **24a–c** and **25a–e**.

*Inhibitor*	*Ki* (nM)	*IC*_50_ (µM)
***23a***	0.82	>1
***23b***	18	>1
***23c***	92.8	>1
***23d***	48.8	>1
***24a***	0.58	0.180
***24b***	1.53	0.381
***24c***	0.63	>1
***25a***	0.014	0.005
***25b***	0.65	0.056
***25c***	0.014	0.005
***25d***	0.025	0.024
***25e***	0.012	0.003
*Darunavir*	0.016	0.003

**Table 7:** Antiviral evaluation of derivative **25e** on a panel of multidrug-resistant HIV-1 variants.

*EC*_50_* ± SD* (µM)			
*Virus*	Aprenavir	Darunavir	25e
*HIV-l104pre(wt)*	0.037 ± 0.0003	0.0035 ± 0.0004	0.0048 ± 0.0002
*HIV-IMDR/B*	0.044 ± 0.13 (12)	0.028 ± 0.006 (8)	0.036 ± 0.003 (8)
*HIV-lMDR/C*	0.38 ± 0.11 (10)	0.028 ± 0.006 (8)	0.0029 ± 0.0002 (1)
*HIV-IMDR/G*	0.398 ± 0.009 (11)	0.023 ± 0.001 (7)	0.0047 ± 0.0007 (1)

**Table 8:** Structure-activity relationships and in vivo profiling of strict biphenyl antiviral agents.

Lead Chemotype/Modification	Viral Target	Conformational & SAR Rationale	Preclinical/Clinical Profile
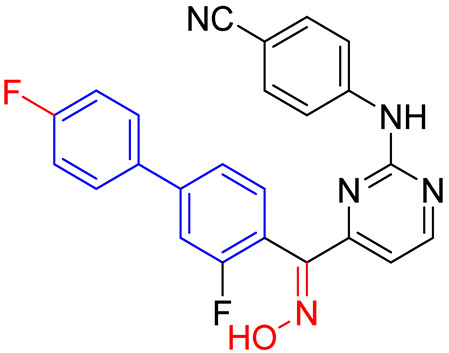 Oxime-Biphenyl-DAPYs & Pyridones	HIV-1 RT (WT and Mutants)	*Ortho*-substitutions widen the dihedral angle to dynamically fill hydrophobic RT sub-pockets; halogens increase electronic affinity.	High Selectivity Index (>24,000). Exceptionally low cytotoxicity in MT-4 cells.
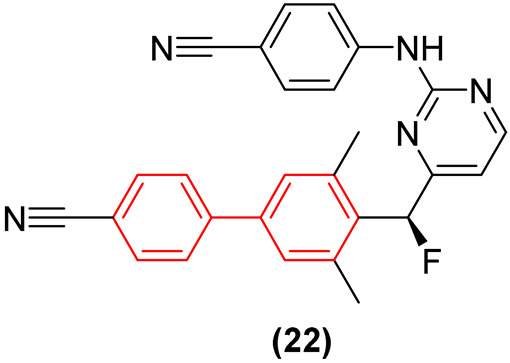 Halomethylene-Biphenyl-DAPYs	HIV-1 RT (Metabolic Shielding)	Halogenation of the linker restricts torsional flexibility into a “horseshoe” shape and bioisosterically protects against metabolic oxidation.	Significantly improved PK profiles (F = 49.5%) and high in vivo safety (LD_50_ > 2 g/kg).
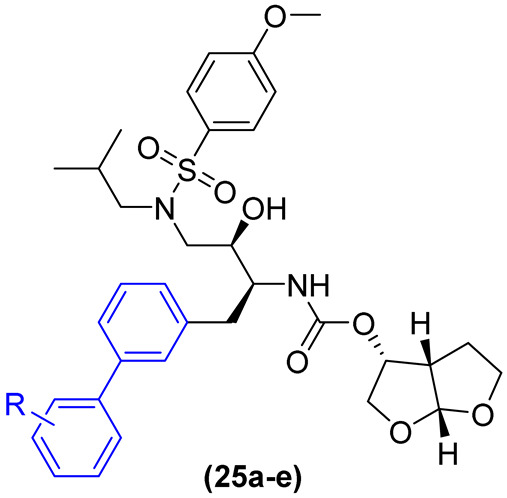 Biphenyl-substituted Darunavir analogs	HIV-1 Protease (MDR strains)	The extended biphenyl framework replaces the benzyl ring to maximize van der Waals contacts in the mutated S1′ subsite.	Sub-nanomolar enzymatic inhibition (Ki = 0.012 nM) across multidrug-resistant HIV-1 variants.
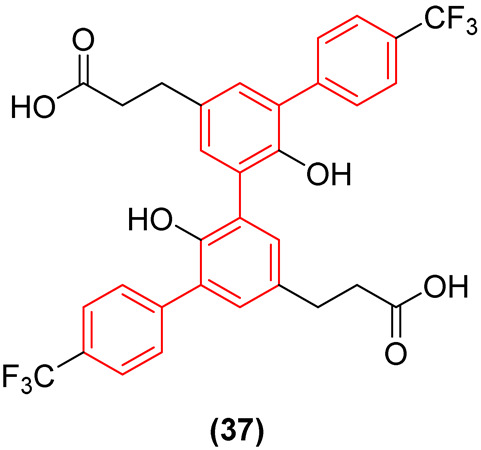 Dual-Activity Biphenyls	SARS-CoV-2 Mpro (3CLpro)	The inclusion of an additional phenyl ring doubles the biphenyl topology, allowing the extended hydrophobic core to deeply penetrate the S4 pocket while maximizing electrostatic interactions via halogenation.	Potent 3CLpro enzymatic inhibition (IC_50_ = 0.15 µM), strong cellular antiviral activity, and dual anti-bladder cancer efficacy.
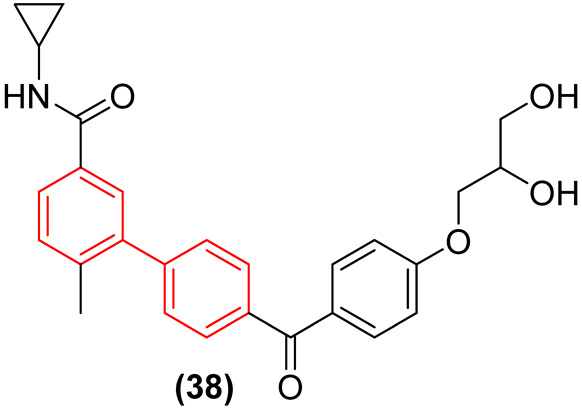 Biphenyl-based p38 MAPK Inhibitor	Hepatitis B Virus (HBV)	The strict biphenyl wedges selectively into the host kinase allosteric hydrophobic pocket, inhibiting virus-induced host signaling.	High barrier to resistance (host-directed therapy) and minimal cytotoxicity in mammalian models.

**Table 9:** Comprehensive Cross-Sectional SAR, Mechanistic Insights, and In vivo Profiles of Biphenyl-Based Antibacterial Agents.

Lead Chemotype/Modification	Target Mechanism & Primary Pathogen	Key Biphenyl SAR Contribution & Conformational Impact	Preclinical/In Vivo Profile
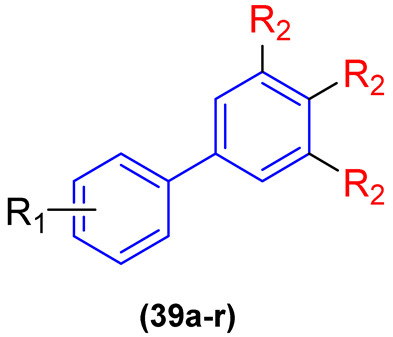 Polyhydroxylated Biphenyl Phytoalexins	Bacterial Membrane Disruption (MRSA, *E. faecalis*)	Strong electron-withdrawing A-ring (-CF_3_) combined with B-ring polyhydroxylation provides an optimal push-pull electronic distribution.	High translation potential against entrenched Gram-positive resistance.
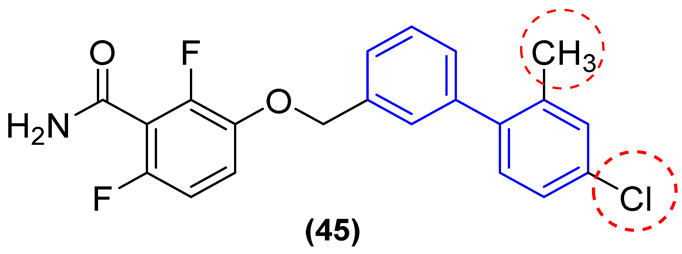 Biphenyl Benzamides	Cell Division—FtsZ Inhibition (*B. subtilis*, MRSA)	Scaffold hopping from benzene to a strict biphenyl exponentially increases target affinity; functions as a highly selective bactericidal agent.	Exceptional in vitro selectivity; no cytotoxicity in mammalian Vero cells.
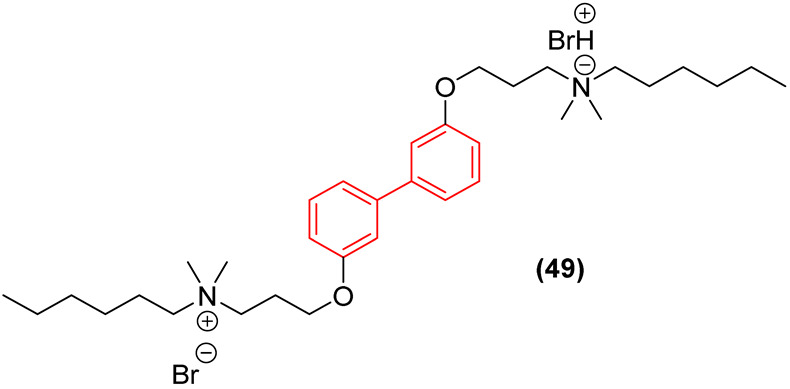 Biaromatic Core-Linked Quaternary Ammoniums	Bacterial Membrane Disruption (MRSA)	The 3,3′-biphenyl linkage acts as a rigid spacer preventing hydrophobic collapse, ensuring selective binding to PG/CL.	High stability in human plasma and exceptionally low hemolytic toxicity.
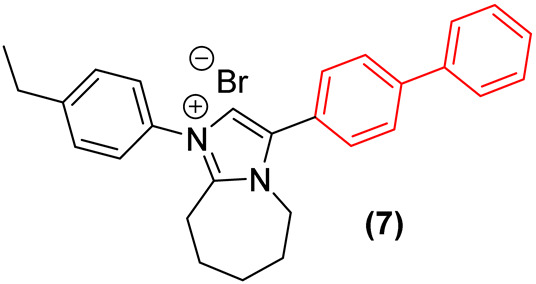 Biphenyl-Imidazo[1,2-a]azepines	Lipophilic Pathogen Targeting (MRSA)	A bulky biphenyl substituent at position 3 provides essential hydrophobic contacts; strictly demands a 7-membered azepine ring.	Exhibits minimal hemolysis of human erythrocytes.
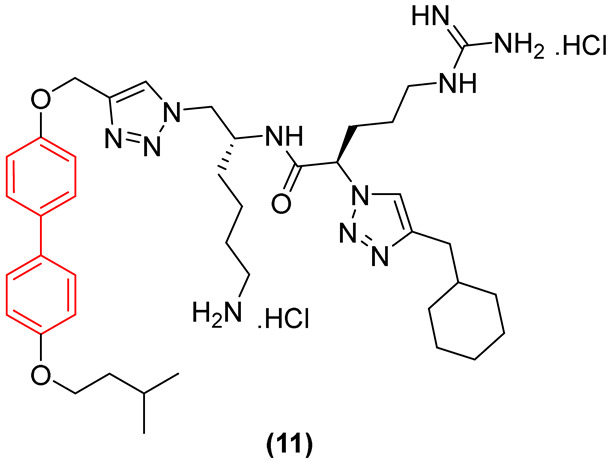 Positional Isomers of Biphenyl Peptidomimetics	Outer Membrane Penetration (*P. aeruginosa*, *E. coli*)	The 3,2′-disubstitution topologically optimizes facial amphiphilicity to overcome Gram-negative outer barriers compared to 2,2′-isomers.	Significant reduction in mammalian cell hemolysis.
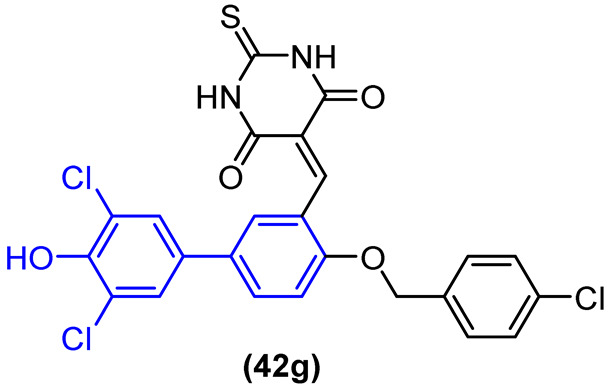 Thiobarbiturate-Biphenyls (e.g., Dichlorophenol derivative)	Virulence Factor—MptpB Inhibition (*M. tuberculosis*)	Halogenation (Cl, CF3O) on the biphenyl ring enhances target engagement within the P1 pocket of MptpB in the host cytoplasm.	IC_50_ = 1.18 µM; targets intracellular virulence without inducing Vero cell cytotoxicity.
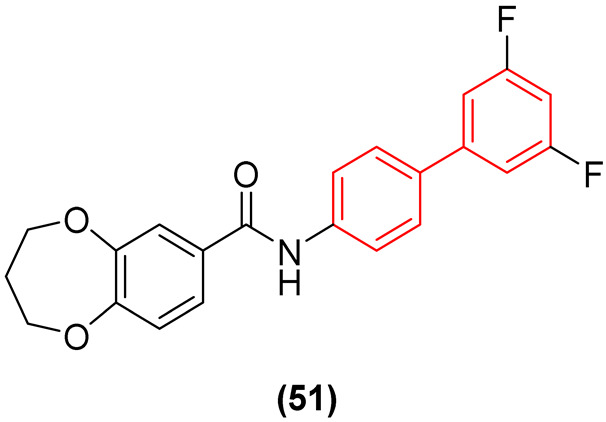 F- and CF_3_-Substituted Biphenyls	Type II Fatty Acid Synthesis—FabH Inhibition (*E. coli*, *S. aureus*)	*Ortho*/*meta* bis-fluorination restricts dihedral rotation for an exact fit within the FabH active site, enhancing affinity and stability.	IC_50_ = 4.1 µM against FabH; broad-spectrum activity with minimal in vitro cytotoxicity.
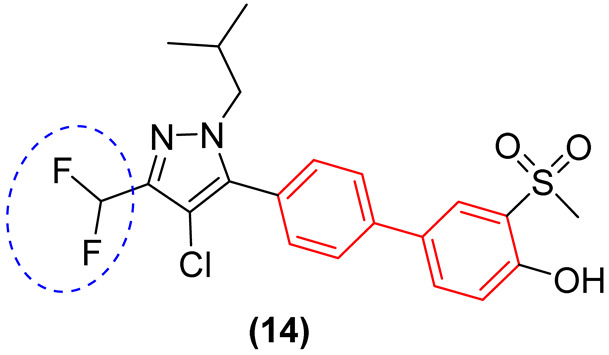 Biphenyl-Pyrazoles	Lipophilic Pathogen Penetration (*P. mirabilis*)	Tethering the 1,1′-biphenyl system at the 1- or 5-position of the pyrazole core maximizes lipophilicity for Gram-negative bacilli penetration.	MIC = 15.62 µg/mL against *P. mirabilis*; significant inhibition of biosynthetic pathways.
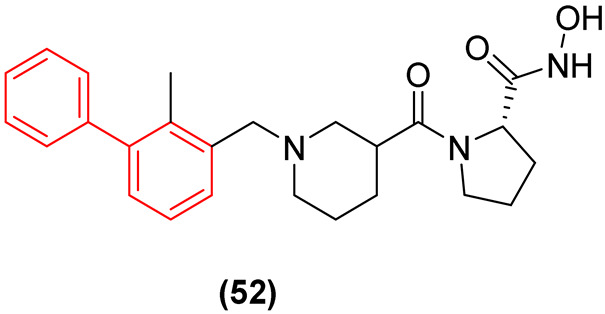 Biphenyl Hydroxamic Acid Hybrids	LpxC/PD-L1 Dual Inhibition (*K. pneumoniae*, *P. aeruginosa*)	A strict biphenyl PD-L1 dimerizer is fused with an LpxC-targeting hydroxamic acid, halting LPS synthesis and restoring host immunity.	Elicited a 100% survival rate with zero adverse systemic events in murine in vivo models.

**Table 10:** Antitrypanosomal activity and in vitro cytotoxicity of best furanochalcone-biphenyl hybrids.

*Compound*	*Cytotoxicity* *LC*_50_ µM *(U-937 Cells)*	*Antitrypanosomal Activity* *EC*_50_ µM	*SI*
***62a***	16.34 ± 0.12	17.81 ± 0.75	0.92
***62b***	15.82 ± 0.15	13.59 ± 1.23	1.16
***62c***	15.81 ± 0.39	15.61 ± 1.71	1.01
***62d***	16.04 ± 0.26	15.71 ± 1.86	1.02
***63a***	18.99 ± 0.98	18.15 ± 0.54	1.05
***63b***	19.44 ± 1.40	16.79 ± 2.28	1.16
***63c***	15.65 ± 0.17	15.53 ± 1.85	1.01
***63d***	15.61 ± 0.32	12.59 ± 1.01	1.24
***63e***	15.74 ± 0.23	10.52 ± 0.23	1.50
***63f***	17.77 ± 0.46	13.42 ± 0.39	1.32
***63h***	17.54 ± 0.22	30.41 ± 5.46	0.58
*BNZ*	687.80 ± 16.14	40.3 ± 6.92	17.0

**Table 11:** Comprehensive Structure-Activity Relationship (SAR) trends and clinical translation profiling of biphenyl-based antiparasitic agents.

Lead Chemotype	Pathogen	Role of the Strict Biphenyl Core (SAR Insight)	Pharmacological Outcome & Pharmacokinetic/Toxicity Limitations
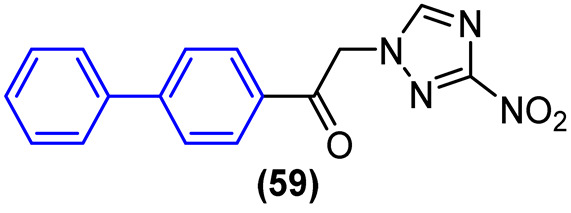 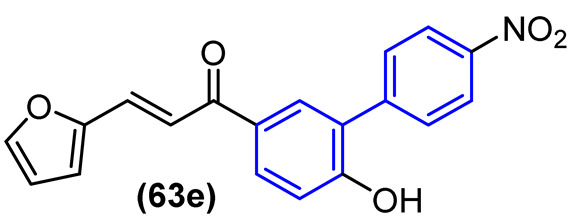 Nitroreductase/CYP51 inhibitors & Furanochalcone hybrids	*Trypanosoma cruzi* & *T. brucei*	Replaces simple/difluorobenzenes to vastly improve the spatial fit within deep hydrophobic enzymatic active sites. Promotes macrophage penetration.	Exceeds the efficacy of clinical standards (e.g., benznidazole) against intracellular amastigotes. However, highly lipophilic derivatives exhibited moderate cytotoxicity (low Selectivity Index).
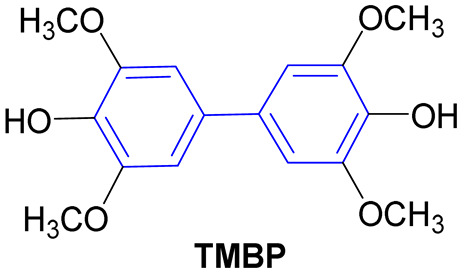 TMBP (Tetramethoxy-biphenyl-diol)	*Leishmania amazonensis*	The polyoxygenated, sterically hindered biphenyl scaffold actively localizes to the mitochondria, inducing severe reactive oxygen species (ROS) generation.	Triggers direct protozoal apoptosis. Demonstrates a high Selectivity Index and proves to be non-hemolytic to mammalian erythrocytes.
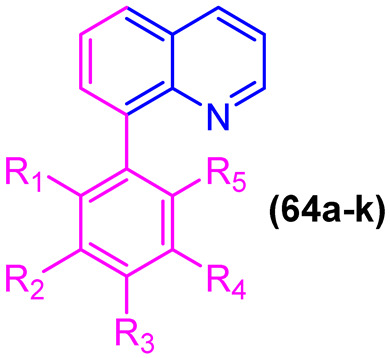 Quinoline-biphenyl hybrids	*Plasmodium falciparum* (WT and Drug-Resistant strains)	The rigid biphenyl acts as a highly lipophilic bridge linking dual pharmacophores, improving binding affinity to multiple targets simultaneously (hybridization strategy).	Produces broad-spectrum antiprotozoal activity. Modest aqueous solubility requires careful formulation or further structural functionalization.
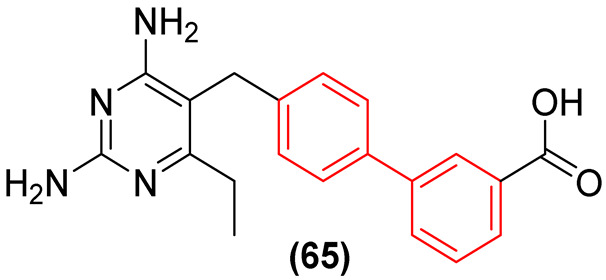 2,4-Diaminopyrimidine-biphenyl carboxylates	*Plasmodium falciparum* (QM Pyrimethamine-Resistant)	Provides severe conformational restriction. The scaffold acts as a precise geometric vector to anchor the drug into the PABA pocket, while stabilizing against CYP450 metabolism.	Sub-nanomolar enzymatic inhibition and excellent metabolic stability. Limitation: The profound rigidity and lipophilicity severely reduce kinetic solubility at physiological pH, causing a massive gap between enzymatic affinity and cellular efficacy.
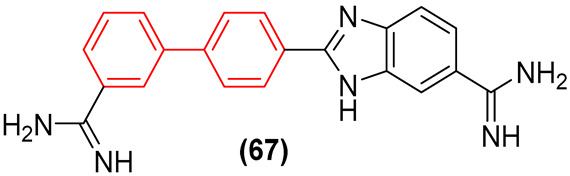 Dicationic biphenyl benzimidazole diamidines	*Trypanosoma brucei rhodesiense*	The linear 4,4′-biphenyl axis acts as a rigid spacer, perfectly aligning terminal dicationic groups to selectively target the minor groove of kinetoplast AT-rich DNA.	Exceptional in vivo translation. Achieved sterile cures in murine models at extremely low doses (0.20 mg/kg).

## Data Availability

No new data were created or analyzed in this study.
